# Electronic structure and reactivity of nickel(i) pincer complexes: their aerobic transformation to peroxo species and site selective C–H oxygenation†
†Electronic supplementary information (ESI) available. CCDC 1439102–1439106. For ESI and crystallographic data in CIF or other electronic format see DOI: 10.1039/c5sc04644k


**DOI:** 10.1039/c5sc04644k

**Published:** 2016-02-11

**Authors:** Christoph A. Rettenmeier, Hubert Wadepohl, Lutz H. Gade

**Affiliations:** a Anorganisch-Chemisches Institut , University of Heidelberg , Im Neuenheimer Feld 270 , 69120 Heidelberg , Germany . Email: lutz.gade@uni-hd.de

## Abstract

The study is aimed at a deeper understanding of the electronic structure of the T-shaped nickel(i) complex [Lig_iPr_(iso)Ni] (**1b**), bearing the iso-PyrrMeBox (bis(oxazolinylmethylidene)pyrrolidinido) pincer ligand, and its CO adduct [Lig_iPr_(iso)Ni(CO)] (**2b**) as well as to provide insight into the mechanism of autoxidation of the different nickel peroxo species of this ligand type. CO was found to react reversibly with complex **1b** resulting in the corresponding CO adduct **2b**. The EPR data as well as the results of DFT modeling revealed significant differences in the electronic structure of **1b** and **2b**. Reaction of [Lig_Ph_(iso)Ni] and [Lig_iPr_(iso)Ni] (**1a** and **b**) with dioxygen yielded the 1,2-μ-peroxo complexes [Lig(iso)NiO]_2_**3a** and **b** which reacted with hydrogen peroxide to give the hydroperoxo complexes [Lig(iso)NiOOH] **5a** and **b**. Thermal aerobic decomposition of the peroxo species **3a** and **5a** in the presence of O_2_ led to a C–H activation of the ligand at the benzylic position of the oxazoline ring forming diastereomeric cyclic peroxo complexes **6** and **6′**. For the 1,2-μ-peroxo complex **3b** the autoxidation of the pincer in the absence of O_2_ occurred at the tertiary C–H bond of the ^i^Pr-group and led to a selective formation of the terminal hydroxo complex [Lig_iPr_(iso)NiOH] **7b** and the cyclic alkoxy complex **8** in equimolar quantities, while the corresponding cyclic peroxo species **9** was formed along with **7b** in the presence of oxygen. Whether or not O–O bond cleavage occurred in the generation of **9** was established upon performing labeling experiments which indicate that the transformation does not involve an initial O–O bond cleaving step. Based on these observations and a series of stoichiometric transformations a tentative proposal for the processes involved in the anaerobic and aerobic decomposition of **3b** has been put forward. Finally, the nickel(ii) methyl complex [Lig_Ph_(iso)NiMe] **14** reacted with O_2_ to give the methylperoxo complex [Lig_Ph_(iso)NiOOMe] **15** which slowly converted to a mixture of near equal amounts of the formato and the hydroxo complexes, [Lig_Ph_(iso)NiOOCH] **16** and [Lig_Ph_(iso)NiOH] **7a**, along with half an equivalent of methanol. The formato complex **16** itself decomposed at elevated temperatures to CO_2_, dihydrogen as well as the nickel(i) species **1a**.

## Abstract 2

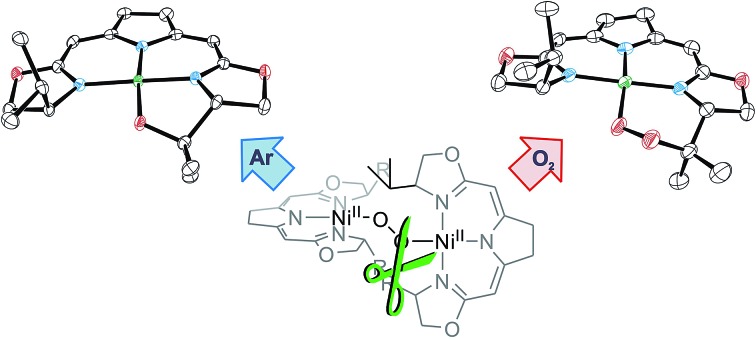
The dinuclear nickel peroxo complexes undergo autoxidation giving a cyclic alkoxo or peroxo complex in the presence or absence of O_2_, respectively.

## Introduction

Redox active nickel containing enzymes such as [Ni/Fe] hydrogenase,^1^,^2^ acetyl CoA synthase (ACS),^3^,^4^ CO dehydrogenase and methyl coenzyme M reductase (MCR)^5^,^6^ are mainly found in biological systems existing under anaerobic conditions where these nickel enzymes play a crucial role in methanogenesis.^7^,^8^ In contrast to the diverse redox chemistry of nickel enzymes under anaerobic conditions, the importance of nickel in enzymatic, aerobic redox processes is limited to those of nickel containing superoxide dismutase (SOD).^9^,^10^ Whether nickel oxygen intermediates are involved in the catalytic cycle is currently under debate.^11^,^12^

Reactive nickel oxo, peroxo and superoxo intermediates have been synthesized in recent years *via* the direct reaction of nickel(i) complexes with oxygen.^13^–^21^ Such nickel oxygen species were found to be capable to intra-22–28 and intermolecularly^13^,^29^–^33^ activate C–H bonds.

**Fig. 1:**
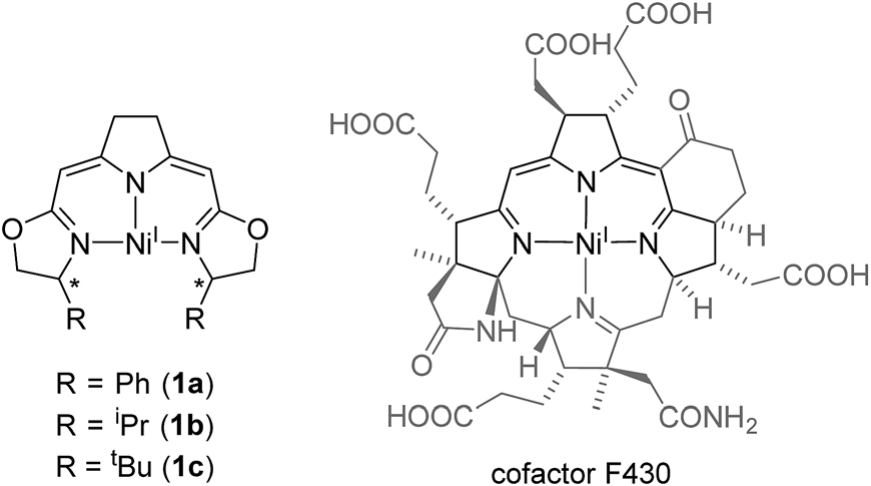
Comparison of nickel(i) complexes bearing the monoanionic iso-PyrrMeBox pincer ligands (left) and the hydrocorphin system found in cofactor F430 (right).^6^

The monoanionic iso-PyrrMeBox ligands developed in our group^34^,^35^ bear key structural features found in the hydrocorphin system of cofactor F430 of methyl coenzyme M reductase (Fig. 1). Both ligand systems contain an almost identical delocalized 10-electron-π-system involving three nitrogen donor atoms and unsaturated carbon-linkers which interacts with the central metal ion upon its coordination.^36^

While both the F430-hydrocorphin and the iso-PyrrMeBox ligands are capable of stabilizing nickel(i) complexes (Fig. 1), the absence of the fourth donor function of the iso-PyrrMeBox ligand leads to complexes with an available coordination site in the ligand plane in close proximity to the chiral centers of the oxazoline rings. This combination of electronic and structural features makes the iso-PyrrMeBox ligand an ideal candidate for the systematic investigation of its reactivity in catalysis and at the same time opens up the possibility to isolate reactive intermediates.^37^–^39^

Recently, we reported the synthesis of the first nickel hydroperoxo complex characterized to date as well as rare nickel-1,2-μ-peroxo complexes using this iso-PyrrMeBox ligand.^37^ The current work is aimed at a deeper understanding of the electronic structure of the T-shaped nickel(i) pincer complexes, how it is modified by the coordination at the fourth in-plane coordination site and to provide insight into the mechanism of autoxidation of the different nickel peroxo species of this ligand type.

## Results and discussion

### Coordination at the free coordination site of the T-shaped nickel(i) complexes and its impact on the electronic structure

The electron rich nickel(i) complex **1** showed little tendency to coordinate small molecules such as N_2_ or CO_2_ or classical donor ligands such as THF or N-heterocycles at the available fourth coordination site, and no such derivatives were found to be isolable or even detectible. This observation raised the question whether the occupation of the fourth coordination site by the pyrroline donor in the cofactor F430 is enforced by the macrocyclic hydrocorphin (Fig. 1), thus giving rise to only a minor additional metal ligand stabilization.

**Scheme 1:**
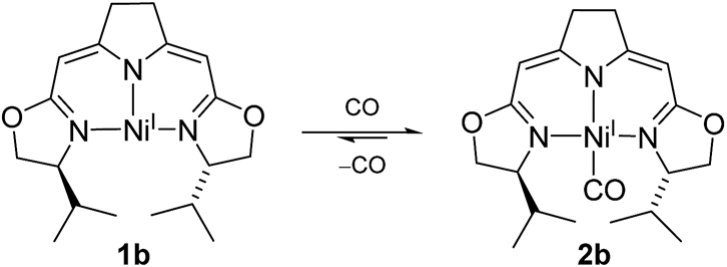
CO dependent equilibrium between nickel(i) complexes **1b** and **2b**.

On the other hand, the strong π-acceptor CO was found to react reversibly with the T-shaped nickel(i) complex **1b** resulting in a CO pressure dependent equilibrium between the threefold-coordinated nickel(i) species **1b** and the corresponding CO adduct **2b** (Scheme 1). Exposure of solutions of **2b** to an evaporated headspace led to the complete loss of CO, reforming the nickel(i) complex **1b**. We note that nickel(i) carbonyl species are believed to be involved in the acetyl-CoA synthesis by ACS as part of the Wood–Ljungdahl pathway in methanogenesis^40^,^41^ and it has been shown recently that PNP pincer complexes were able to mimic a key step, namely the methyl transfer onto the carbonyl species to form the corresponding acetyl complex.^42^

The coordination of CO to the nickel(i) center is accompanied by a change in color from red to green so that the reversible reaction can be monitored by UV/Vis and IR spectroscopy (ESI†). The characteristic band of the CO vibration was found at 1955 cm^–1^ in the IR spectrum (KBr) of the precipitate obtained from the equilibrium mixture at 10 bar CO at –78 °C indicating moderate back-bonding from the nickel(i) center to the CO ligand.

**Fig. 2:**
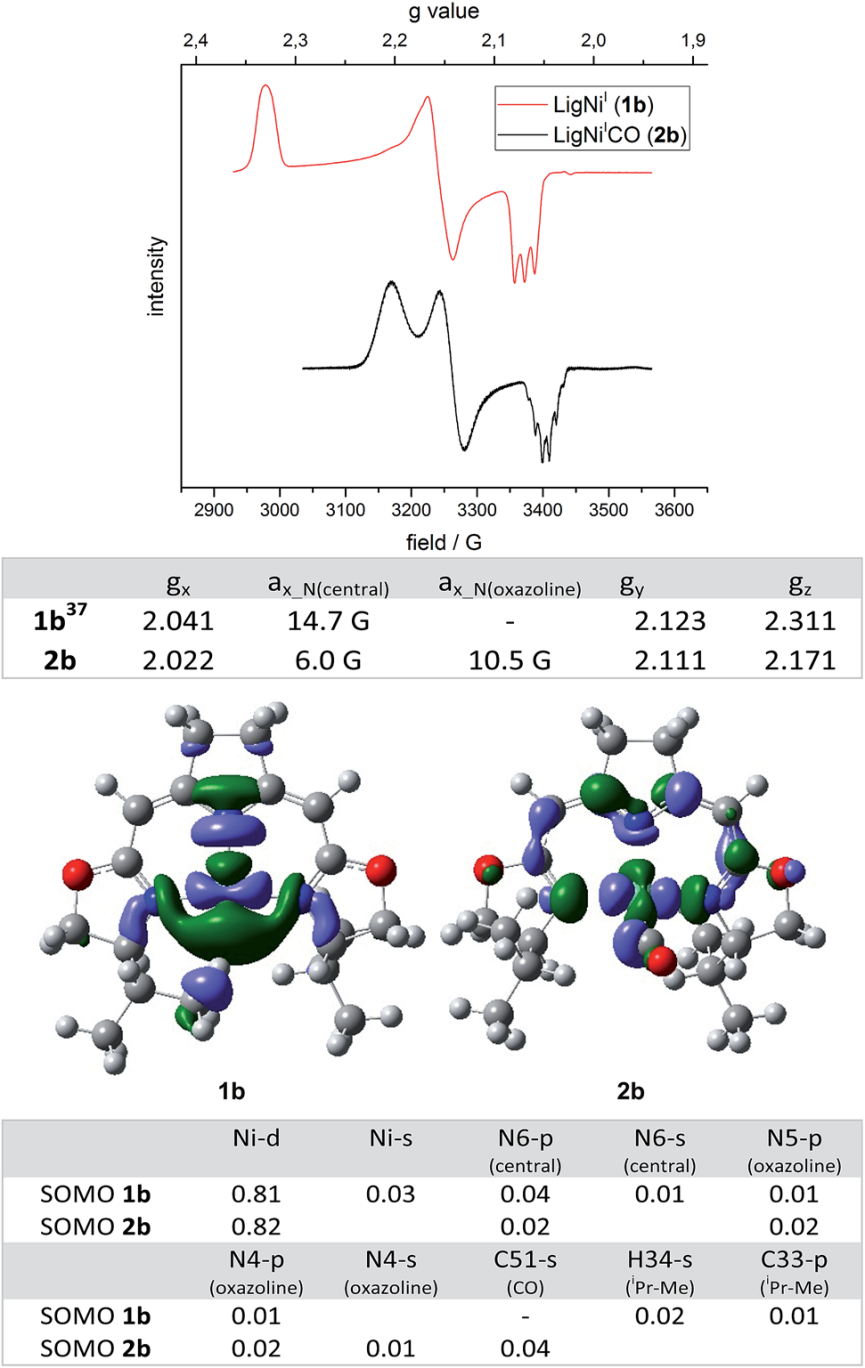
Top: EPR spectra of nickel(i) complexes **1b** and **2b** (963 284 GHz, toluene, 30 K) and the corresponding anisotropic *g* values and coupling constants *a*. Bottom: DFT calculated (ROB3LYP/6-311G(d,p))^45^–^57^ SOMOs (isovalue: 0.04) of nickel(i) complexes **1b** and **2b** and the corresponding atomic contributions (threshold 0.01).

Interestingly, a very similar scenario of a reversible CO coordination to a T-shaped nickel(i) species with analogous electronic changes had been described by Caulton and coworkers for a PNP pincer system bearing two Si atoms in the backbone of the ligand.^43^ In their case and other related pincer complexes, structural data by X-ray analysis of the CO adducts were obtained.^42^,^43^ The main geometric features of this as well as Lee's more recent example are analogous to those of the DFT optimized structure of **2b** (Fig. 2 and ESI†).

In order to obtain insight into the degree to which this occupation of the fourth coordination site by the carbonyl ligand modified the electronic structure of the complex, ESR spectra of **2b** were recorded under a pressure of 10 bar CO. The EPR spectrum of **2b** recorded at 30 K displayed one dominant signal with rhombic symmetry (*g*_x_ = 2.022, *g*_y_ = 2.111, *g*_z_ = 2.171; cofactor F430:^44^*g*_II_ = 2.224, *g*_⊥_ = 2.061) which differed from the resonance of the T-shaped nickel(i) complex **1b** (*g*_x_ = 2.041, *g*_y_ = 2.123, *g*_z_ = 2.311, Fig. 2). In comparison to the EPR signal of the latter a significant shift of the g_z_ value to higher field was observed when the fourth coordination site was occupied by the CO ligand. Furthermore, significant differences in the superhyperfine coupling to the N atoms were observed. While a triplet superhyperfine splitting of the x component of the signal caused by the central pyrrolidine N atom was observed in the EPR spectrum of complex **1b**,^39^ the coupling of all three N donor atoms with the unpaired electron is resolved in the case of the CO adduct **2b** leading to a more complicated coupling pattern (Fig. 2). Assuming *C*_2_-symmetry for **2b** (which is slightly broken by the out-of-plane-coordination mode of the CO ligand), coupling to the central N-atom (N_(central)_) as well as to two (near)-equivalent oxazoline N-atoms (N_(oxazoline)_) accounts for a maximum multiplicity of 15 (triplet of quintet). Due to the different superhyperfine coupling constants at hand, however, superpositioning leads to the pattern that is actually observed experimentally which could be successfully simulated (values are given in Fig. 2, the simulated spectrum is depicted in the ESI†).

The superhyperfine splitting in the EPR spectra of **1b** and **2b** are well reflected in the results of restricted open shell DFT calculations. The isotropic Fermi contact couplings indicate a significantly higher spin density at the two oxazoline N cores in the CO adduct than in the T-shaped nickel(i) complex [(**1b**) *a*_N(oxazoline)_ 2.4 G and 2.4 G, *a*_N(central)_ 10.2 G (**2b**); *a*_N(oxazoline)_ = 9.5 G and 8.7 G, *a*_N(central)_ = 6.7 G]. Both complexes are best described as nickel(i) species (calc. Mulliken spin densities: (**1b**) Ni 0.849; (**2b**) Ni 0.821) in which the unpaired electron resides in molecular orbitals with dominant *d*_*x*^2^–y^2^_ character. The SOMOs of complexes **1b** and **2b** are depicted in Fig. 2 along with the major atomic contributions to each orbital.

The arrangement of the ^i^Pr-substituent found in the modulated minimum structure of **1b** causes one of the methyl C–H moieties to be located in close proximity to the nickel(i) center (DFT: *d*(C–**H**···Ni) = 2.232 Å, *d*(**C**–H···Ni) = 3.097 Å, *d*(C–H) = 1.097 Å, X-ray:^58^*d*(C–**H**···Ni) = 2.38(3) Å (refined H-atom))^59^ In this set-up the C–H group appears to be interacting with the nickel(i) center with a considerable amount of spin density located at the C–H bond (Fig. 2). Indeed, the corresponding C–H bond is slightly elongated compared to the residual average over both ^i^Pr-substituents (*d*(C–H)_av_ = 1.094 Å with *σ* = 0.001 (DFT)). However, from an energetic perspective the orientation of the ^i^Pr-substituent is mainly a result of minimizing steric repulsion between vicinal groups. Therefore, a staggered conformation for all dihedral angles of the substituent is preferred (ESI†). In contrast to **2b** and all other fourfold coordinated complexes of this type analyzed so far, the available coordination site in **1b** allows an ^i^Pr-substituent to adopt a conformation in which one of the methyl groups is located in the open coordination site at the nickel center. The same orientation is observed in the minimum structure of the metal-free ^i^Pr-oxazoline itself with only small differences in the rotational barriers (RB3LYP/6-311(d,p), ESI†). A possible effect on the H NMR shifts was not observed and is expected to be very small due to the rapid exchange of all 12 (6 + 6) methyl proton signals of the ^i^Pr-group even at low temperature. Furthermore, no superhyperfine coupling to the proton is resolved in the EPR spectrum of **1b** (Fig. 2, Fermi contact coupling C 5.0 G, H 1.4 G).

Occupation of the fourth coordination site in the plane of **2b** by the π-acceptor CO thus leads to a significant change in the electronic properties of the complexes. While the *g* tensor of **1b** is highly anisotropic the occupation of the fourth coordination site reduces the anisotropy significantly towards an axial symmetry as observed for the cofactor F430.^44^ A main feature of the T-shaped nickel(i) complex **1b** is the high amount of unpaired electron density at the empty coordination site of the nickel center, which makes the system an attractive candidate for the activation of small molecules/C–X bonds and/in catalysis. This also renders such three-coordinate Ni complexes interesting objects of study for the interaction with O_2_ and resulting oxidation and autoxidation processes.

### Synthesis of peroxo intermediates by the reaction of nickel(i) with oxygen and their thermal decomposition

**Scheme 2:**
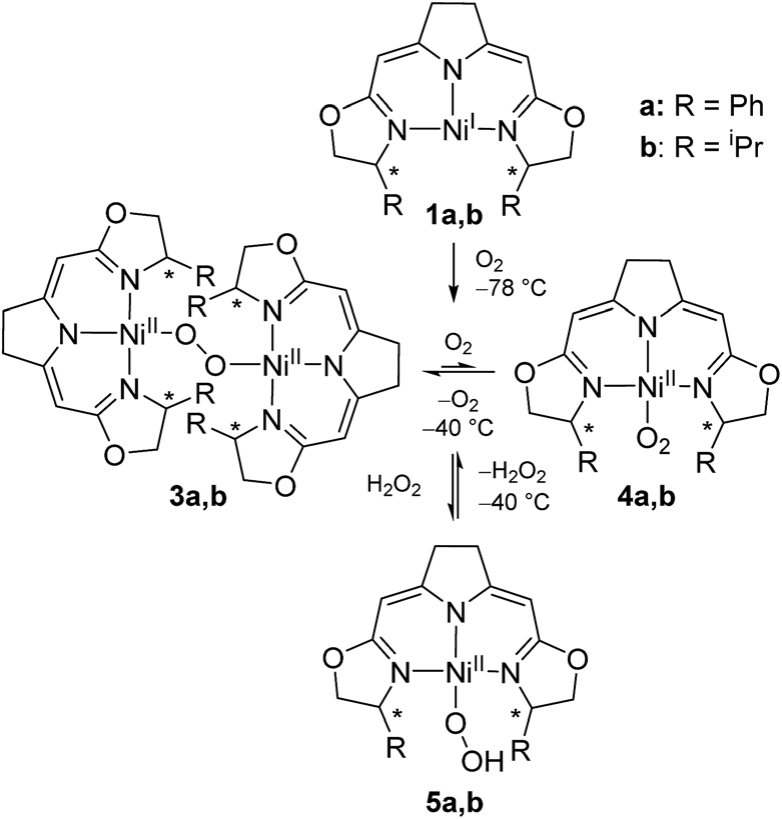
Aerobic formation of the different nickel(ii) peroxo complexes **3a** and **b**, superoxo complexes **4a** and **b** and hydroperoxo complexes **5a** and **b** at low temperatures.

We previously reported the reaction of nickel(i) complexes **1a** and **b** with oxygen at low temperature which leads to 1,2-μ-peroxo complexes **3a** and **b** (Scheme 2).^37^ These bulky dinuclear species were found to exist in an oxygen pressure dependent equilibrium with the corresponding mononuclear paramagnetic superoxo complexes **4a** and **b**.

The addition of hydrogen peroxide to a solution of 1,2-μ-peroxo complexes **3a** and **b** gave the hydroperoxo complexes **5a** and **b**, of which complex **5a**, bearing the phenyl-substituted pincer ligand, could be isolated and structurally characterized by X-ray diffraction.^37^ However, we subsequently observed that the hydroperoxo complex bearing the ^i^Pr-substituted pincer ligand **5b** was unstable in the absence of hydrogen peroxide even at low temperature (–20 °C) and upon attempted isolation its transformation back to the 1,2-μ-peroxo complex **3b** occurred.

### Thermal decomposition of the hydroperoxo complex **5a**

**Scheme 3:**
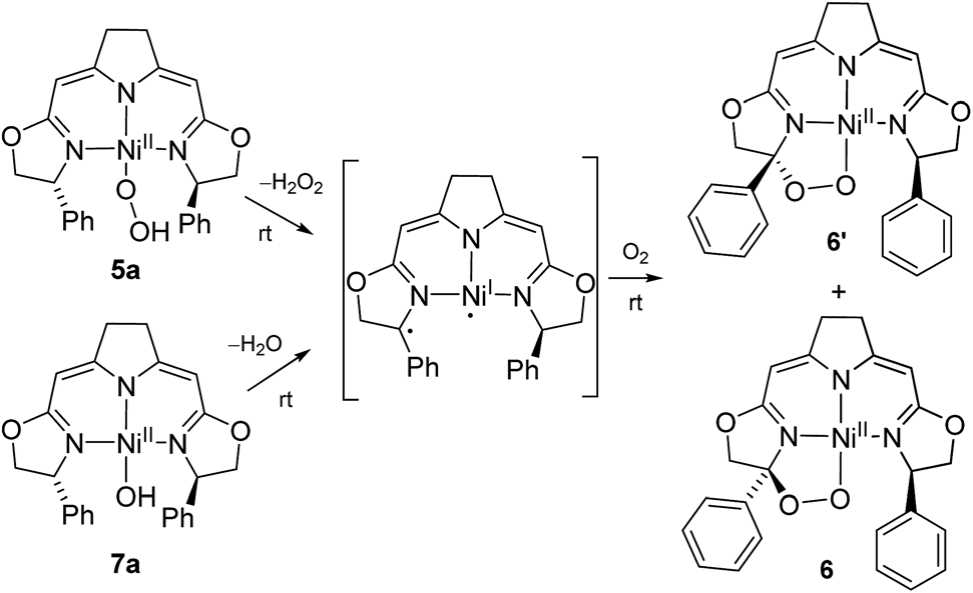
Mechanistic proposal for the formation of the cyclic peroxo complexes **6**/**6′** in the aerobic degradation of the nickel(ii) peroxo and hydroxo complexes **5a** and **7a** at room temperature.

As reported previously,^37^ the thermal aerobic decomposition of hydroperoxo complex **5a** leads to a C–H activation of the ligand at the benzylic position of the oxazoline ring forming cyclic peroxo complexes **6** and **6′** (Scheme 3). The reaction involves the chiral carbon center, and a partial racemization of its configuration takes place during the transformation leading to two diastereomeric species. The loss of stereoinformation is attributed to the intermediate formation of a configurationally labile benzylic radical which then further reacts with O_2_ in a diastereoselective manner to the products **6** and **6′** in a ratio of 1 to 2.3. Furthermore, slow formation of the mixture of the same diastereomers **6** and **6′** occurred when a solution of the hydroxo complex **7a** was stirred under an atmosphere of oxygen.

**Scheme 4:**
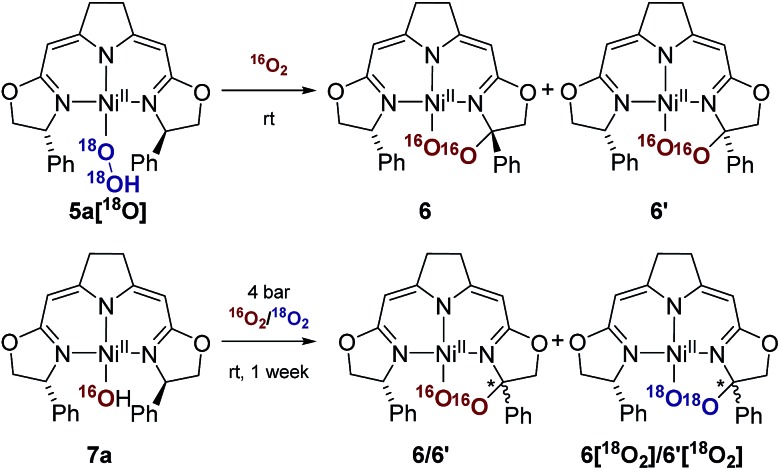
Labeling experiments in the aerobic thermal decomposition of the hydroperoxo and the hydroxo complexes **5a** and **7a** at room temperature.

Since these earlier observations were pertinent to the reactions studied in this work we aimed to substantiate our proposal of a formal O_2_ insertion into a diradical species.^37^ To this end we carried out labeling experiments which showed that, as proposed previously, in both cases the oxygen atoms of the cyclic peroxo species **6** and **6′** originate from the molecular oxygen present in the reaction mixture (Scheme 4). The thermal decomposition of the ^18^O-labeled hydroperoxo complex **5a[^18^O]** in the presence of ^16^O_2_ led exclusively to the homo-isotopologous species bearing two ^16^O atoms. Similarly, when a solution of the hydroxo complex **7a** was held under an atmosphere of ^16^O_2_/^18^O_2_ the homo-isotopologous peroxo species **6** and **6′** were exclusively obtained. Possible scrambling of the O_2_ fragment indicating a homolytic O–O bond dissociation in the course of the transformation was not observed.

### Thermal decomposition of the 1,2-μ-peroxo complexes

**Scheme 5:**
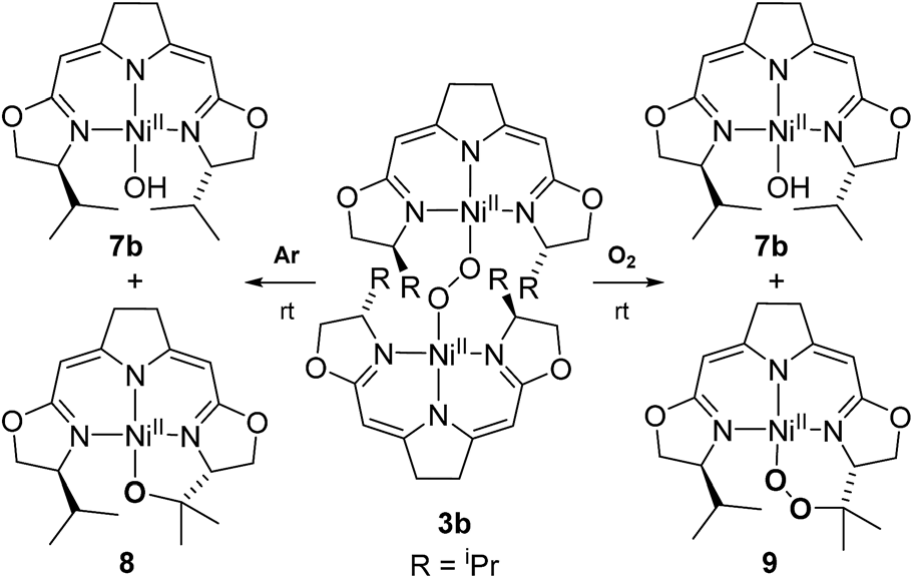
Thermal decomposition of 1,2-μ-peroxo complex **3b** in the absence and presence of oxygen.

In contrast to the hydroperoxo complex **5a** containing the Ph-substituted iso-PyrrMeBox pincer, which could be isolated and fully characterized, the ^i^Pr-substituted hydroperoxo complex **5b** was found to convert to the corresponding 1,2-μ-peroxo complex **3b** even at low temperature. This led us to investigate the thermal autoxidative transformation of this 1,2-μ-peroxo complex **3b** (Scheme 5).

For the 1,2-μ-peroxo complex **3b** the autoxidation of the pincer occurred at the tertiary C–H bond of the ^i^Pr-group instead of the C–H group adjacent to the nitrogen donor of the oxazoline ring. Under anaerobic conditions decomposition of **3b** led to a selective formation of the terminal hydroxo complex **7b** and the cyclic alkoxy complex **8** in equimolar quantities. However, in the presence of O_2_ the corresponding cyclic peroxo species **9** was formed together with the hydroxo complex **7b**, again in a 1 : 1 ratio. We note that a similar reactive behaviour had been observed for the autoxidation of the bis(μ-oxo)dinickel(iii) complex bearing the Me_3_-tpa ligand (tpa = tris(6-methyl-2-pyridylmethyl)amine).^24^,^25^,^27^

**Fig. 3:**
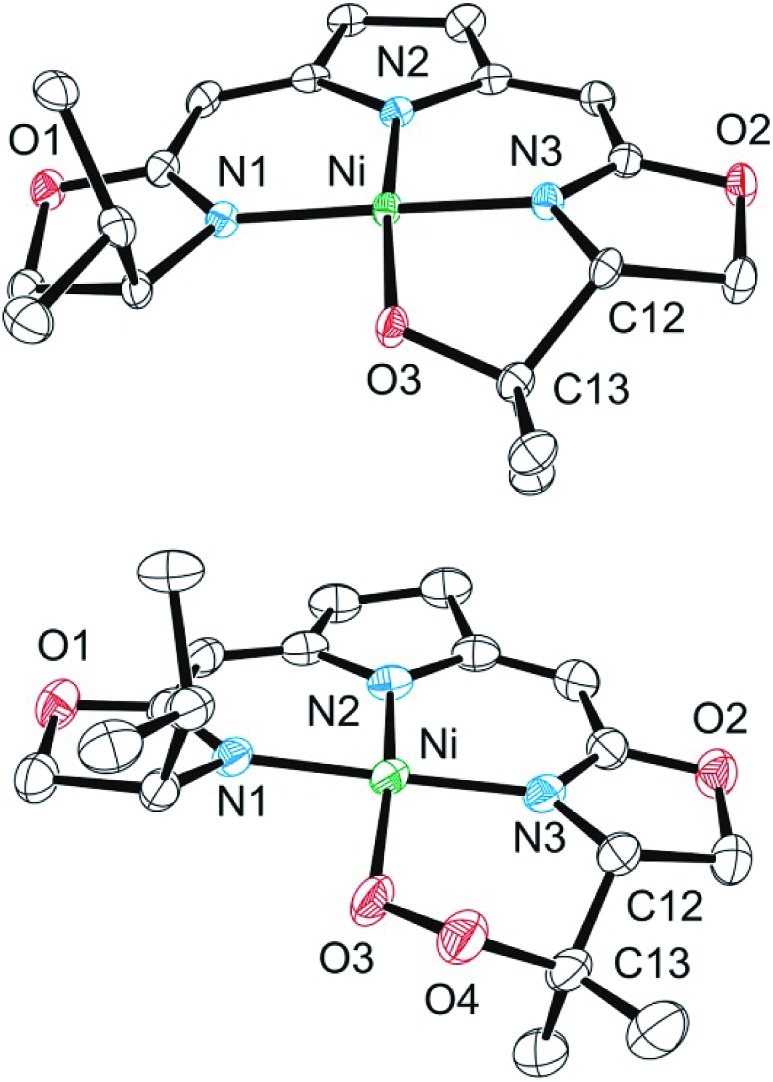
Molecular structure of **8** (top) and **9** (bottom). Hydrogen atoms were omitted for clarity. Selected bond lengths [Å] and angles [°]: **8**: Ni–N(3) 1.811(3), Ni–O(3) 1.857(2), Ni–N(1) 1.868(3), Ni–N(2) 1.903(2), N(3)–Ni–O(3) 84.34(10), N(3)–Ni–N(1) 174.40(11), O(3)–Ni–N(1) 90.90(10), N(3)–Ni–N(2) 90.79(11), O(3)–Ni–N(2) 173.21(10), N(1)–Ni–N(2) 94.19(11); **9**: Ni–O(3) 1.842(3), Ni–N(1) 1.890(3), Ni–N(2) 1.920(3), Ni–N(3) 1.868(3), O(3)–O(4) 1.443(4), O(3)–Ni–N(1) 84.41(12), O(3)–Ni–N(2) 166.68(14), O(3)–Ni–N(3) 92.10(12), N(1)–Ni–N(2) 92.06(13), N(1)–Ni–N(3) 173.17(13), N(3)–Ni–N(2) 92.63(13).

Both metallacycles were isolated and structurally characterized by X-ray diffraction (Fig. 3). In both complexes a distorted square-planar coordination sphere of the nickel center is found. The prominent structural motif of complex **8** is an oxazametallacycle, which adopts a distorted envelope conformation. Due to the geometric constraints of the five-membered ring the N3–Ni–O3 angle [84,34(9)°] is narrowed compared to the ideal square-planar geometry resulting in an in-plane-distortion of the whole coordination sphere around the nickel center.

However, the additional oxygen atom in the cyclic peroxo complex **9** leads to a larger six membered dioxazametallacyle. Thus, a wider N3–Ni–O3 angle [92,10(12)°] is observed resulting in-plane-distortion of the square planar coordination geometry in the opposite sense [sum of the N2–Ni–N3 and N3–Ni–O3 angle 184,73°], as well as a slight out–of plane displacement of the oxygen atom bound to the nickel center. The O–O bond length was found to be 1.443(4) Å.

**Scheme 6:**
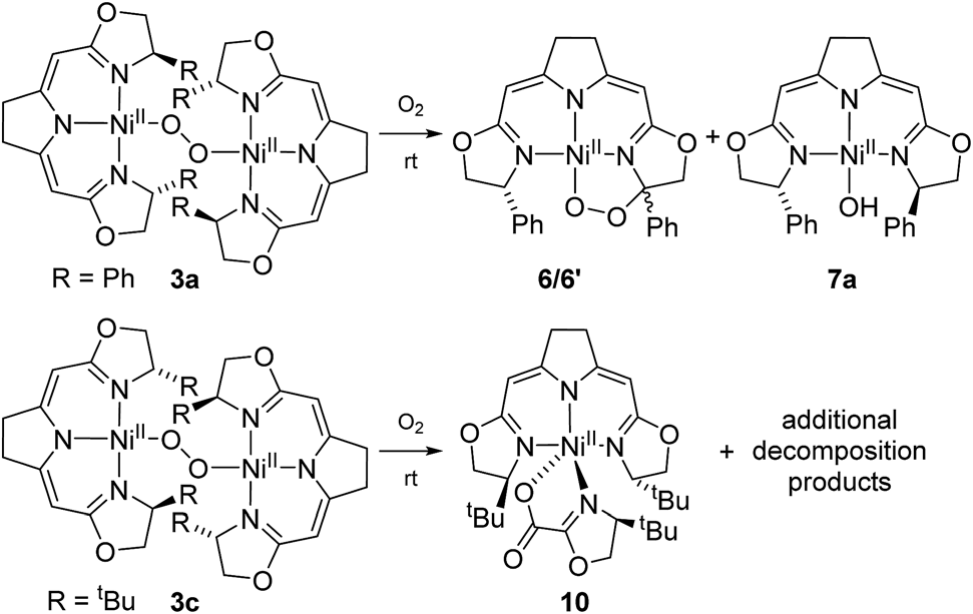
Aerobic autoxidation of 1,2-μ-peroxo complexes **3a** and **3c** at room temperature.

The formation of **9** from the aerobic degradation of the dinuclear peroxo complex **3b** mirrors the generation of the cyclic peroxide **6**/**6′** from hydroperoxo species **5a**. It was therefore of interest to probe for analogous reactivity of the corresponding peroxo species **3a**. Indeed, this dinuclear complex, containing the Ph-substituted pincer cleanly converted to a 1 : 1 mixture of the cyclic alkyl peroxide **6**/**6′** and hydroxo complex **7a** (Scheme 6).

The two nickel 1,2-μ-peroxo complexes **3a** and **b** contain fairly reactive C–H bonds in close proximity to the Ni–O–O–Ni moiety which undergo bond dissociation in the course of their thermal decay. In the absence of such reactive C–H bonds, as in the corresponding nickel peroxo complex **3c** bearing the ^*t*^Bu-substituted iso-PyrrMeBox pincer ligand, non-specific thermal oxidative degradation was observed leading to a mixture of compounds, of which one major product **10** could be isolated and structurally characterized by X-ray diffraction (ESI†). In this complex the bidentate coordination of an oxazolinylcarboxylate to the nickel pincer fragment is observed which is thought to result from the partial oxidative cleavage of the C–C double bond in the backbone of the pincer ligand.

### Mechanistic aspects of the autoxidation of complex **3b**

**Scheme 7:**
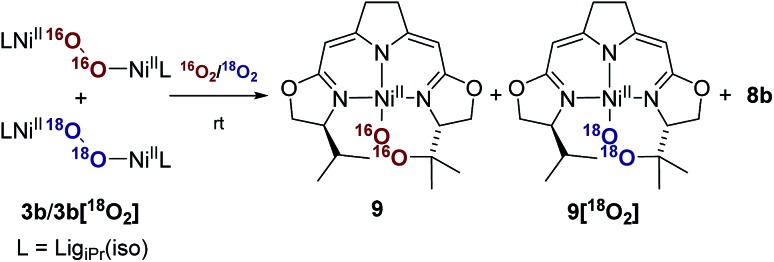
Labeling experiment for the investigation of the oxygen insertion in the course of the thermal decomposition of **3b** applying a mixed atmosphere of ^16^O_2_/^18^O_2_.

Whereas the transformation of **3b** to **7b** and **8** under anaerobic conditions is balanced, the mass balance of the reaction under oxygen atmosphere leading to **7b** and **9** is not readily accounted for. The generation of the cyclic peroxide species **9** raises the question of whether O–O bond cleavage occurred during this transformation or not. In the former case isotope scrambling would be expected upon performing the reaction using the homoisotopically labeled peroxo complexes **3b[^16^O_2_]**/**3b[^18^O_2_]** under an atmosphere of a mixture of homoisotopic ^16^O_2_ and ^18^O_2_ (Scheme 7).

The outcome of this reaction clearly indicated that such scrambling did not occur, *i.e.* that the generation of **9** under O_2_ does not involve an initial O–O bond cleaving step. It was therefore of interest to probe to which extent the presence or absence of external O_2_ influenced the rates of the anaerobic and aerobic transformation of **3b** to the two sets of reaction products depicted in Scheme 5.

**Fig. 4:**
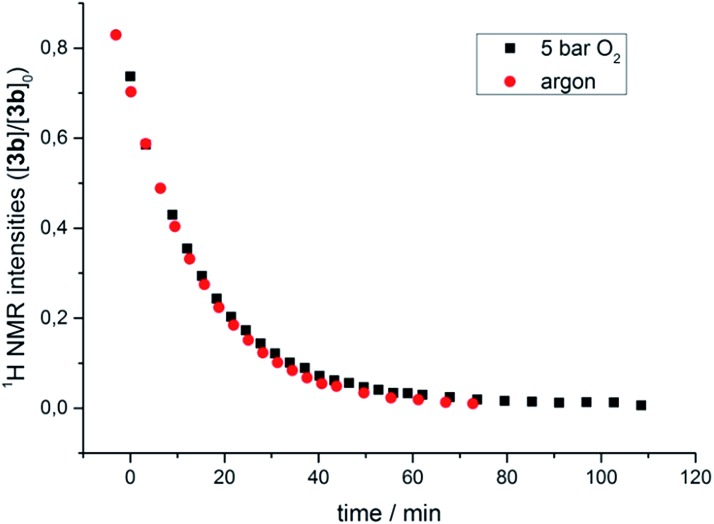
Course of the thermal decomposition of ^i^Pr-substituted μ-1,2-peroxo complex **3b** under argon and in the presence of oxygen (5 bar) in THF monitored by ^1^H NMR spectroscopy.

The transformation of **3b** in solution at room temperature in the presence of an internal standard (1,4-dimethoxybenzene) was monitored by ^1^H NMR spectroscopy, first under an atmosphere of argon and then at 5 bar oxygen pressure. In both cases the data obtained neatly followed the kinetic law of a first order decay. Significantly, oxygen had no effect on the rate of the decomposition (Fig. 4). In the course of the reaction the only species observed in the ^1^H NMR spectra were the starting μ-1,2-peroxo-complex **3b** and the reaction products, while no intermediate could be detected. The identical decay rates under anaerobic and aerobic conditions indicated that **3b** decomposes *via* an initial rate determining step which does not involve an external attack and/or insertion of oxygen.

This observation, along with the labeling study described above, implies a similar early (rate determining) step in the reaction sequence for both cases and contradicts a reaction model for the mass balanced anaerobic conversion of **3b** to **7b** and **8** which would involve an initial O–O bond cleavage, hydrogen abstraction from one iso-propyl group in a pincer ligand (with concomitant formation of the hydroxo complex) and final cyclization of the diradical species generated to give the metallacyclic complex **8**. It also contradicts a decomposition pathway *via* the superoxo species **4** which are in oxygen dependent equilibrium with complexes **3** (Scheme 2).

**Scheme 8:**
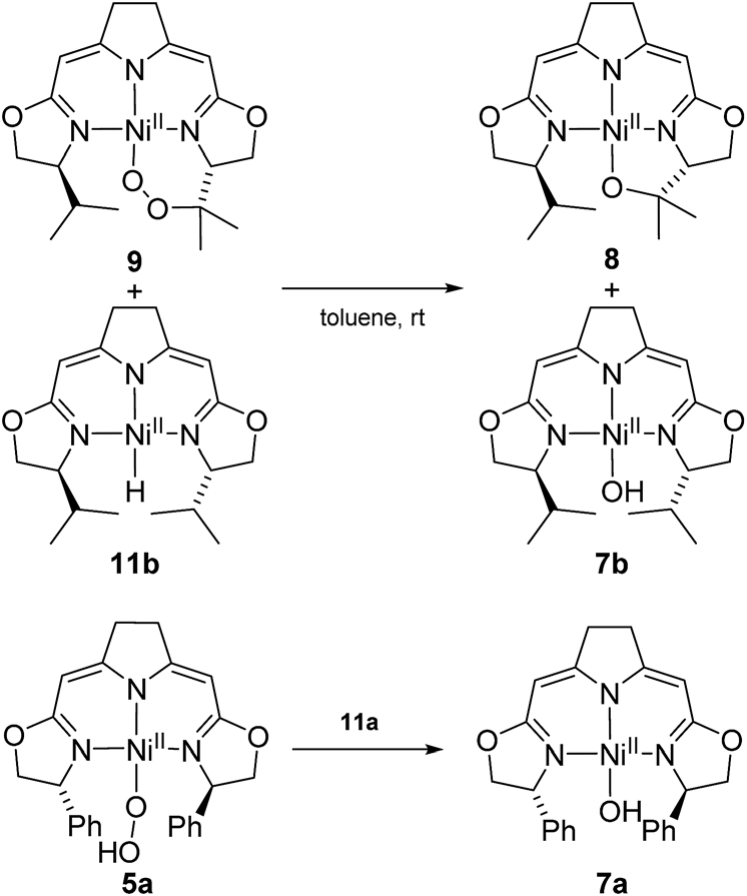
Stoichiometric conversions of cyclic peroxido complex **9** to the alkoxido compound **8** as well as the oxidation of the hydrido complex **11a** to the hydroperoxido complex **5a** and its reduction to **7a**.

Possible alternative mechanistic models could involve the cyclic alkyl peroxo species **9** as key intermediate for both transformations. To probe for this possibility several stoichiometric test reactions were carried out (Scheme 8). Reaction of the cyclic alkyl peroxo complex **9** with an equimolar quantity of nickel hydrido compound **11b**, generated *in situ* by pressurizing a solution of **3b** with H_2_ gave a 1 : 1 mixture of compounds **7b** and **8** as observed in the anaerobic degradation of **3b**. These findings demonstrate that **9** could be a precursor in the formation of **8**, provided that a nickel hydrido species was generated in an earlier step. On the other hand, the hydroperoxo complex **5a** reacted with the Ni–H complex **11a** to give the hydroxo species **7a**.

**Scheme 9:**
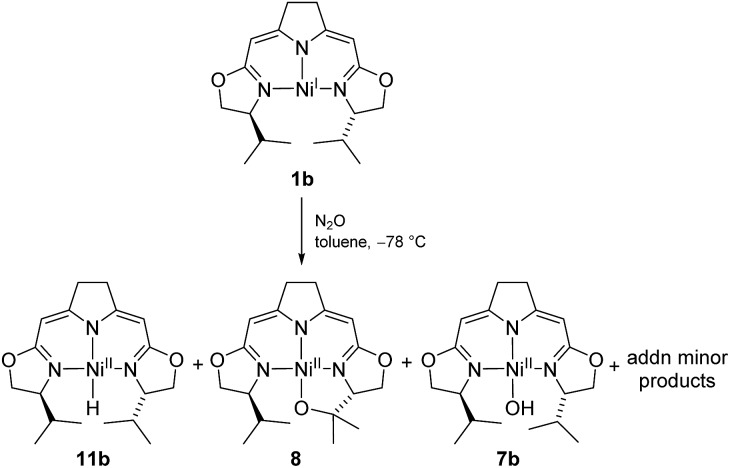
Formation of the hydrido complex **11b** in the reaction of the nickel(i) complex with N_2_O at low temperatures.

These observations may imply that the formation of **8** or **9** as represented in Scheme 5 is dependent on the presence or absence of a hydrido species **11b** generated in the early stages of the autoxidation and which is consumed in the presence of O_2_, a transformation previously reported for palladium and platinum hydrido complexes,^60^–^62^ thus leaving the cyclic alkyl peroxide **9** as isolated from the reaction. The formation of such a hydrido species in an autoxidation process appears counterintuitive, and in fact, could not be proved directly in case at hand. However, we were able to detect such a nickel hydride directly in the reaction of nickel(i) compound **1b** with the oxidant N_2_O at –78 °C indicating that the hydride formation under oxidative conditions may occur (Scheme 9).

**Scheme 10:**
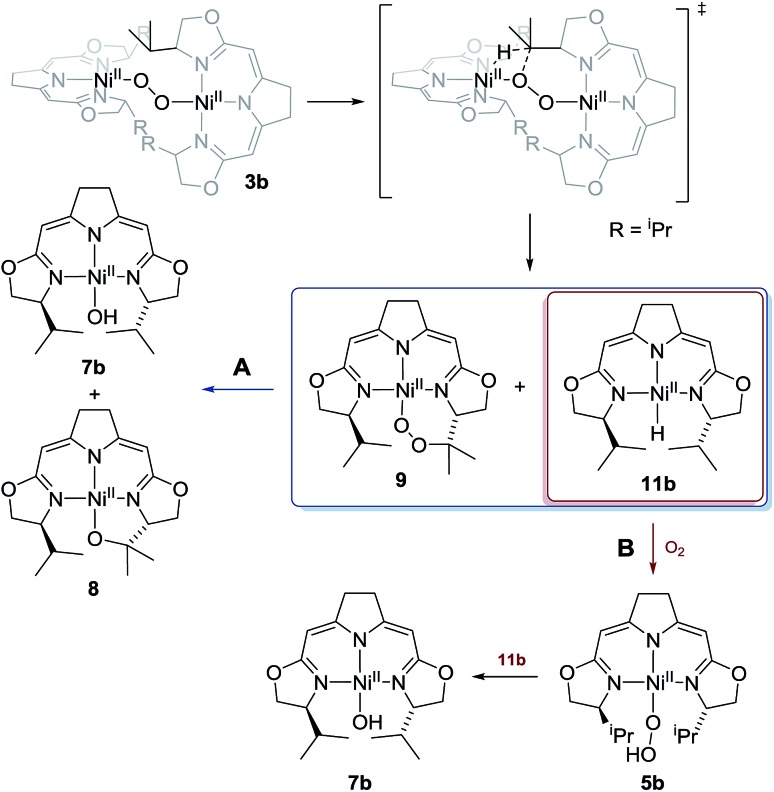
Proposed mechanism for the thermal decomposition in case of the 1,2-μ-peroxo complex **3b** in absence (reaction path A) and presence of oxygen (reaction path B).

Whilst the experimental pieces of evidence gained for this highly reactive and sensitive system do not allow for a complete mechanistic picture, the tentative proposal for the processes involved in the anaerobic and aerobic decomposition of **3b** as represented in Scheme 5 is given above (Scheme 10). The reaction scheme accounts for all of the observations described above.

### Formation and thermal decomposition of a nickel methylperoxo complex

**Scheme 11:**
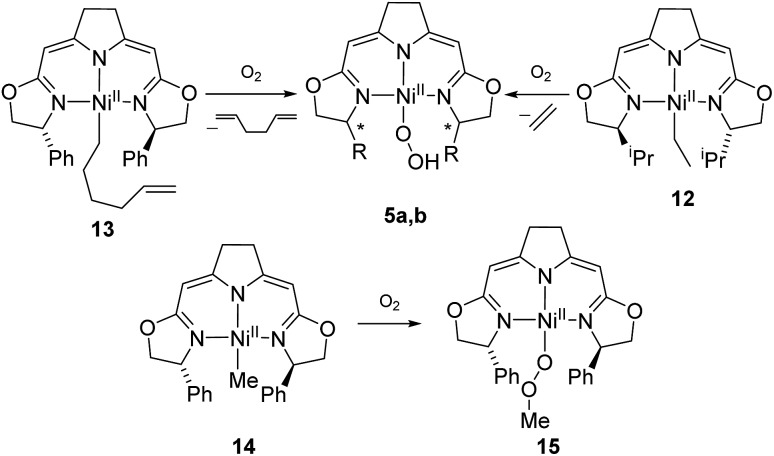
Reaction of alkylnickel complexes with oxygen.

The autoxidation of the pincer ligand in the nickel complexes described above raised the question about the reactivity of their alkyl derivatives towards oxygen. Exposure of a solution of the nickel ethyl complex **12** to an atmosphere of oxygen at –78 °C led to a rapid elimination of ethylene to form the hydroperoxo complex **5b** (Scheme 11), which was subsequently converted to the dinuclear 1,2-μ-peroxo complex **3b** as described above. In order to probe for a possible alkyl radical intermediate in the reaction with oxygen, the hexenyl complex **13** was synthesized and subjected to oxygen at low temperature. However, no rearrangement occurred under these conditions and 1,5-hexadien was the only alkene formed during the reaction indicating that no long-lived radical species were involved.

**Fig. 5:**
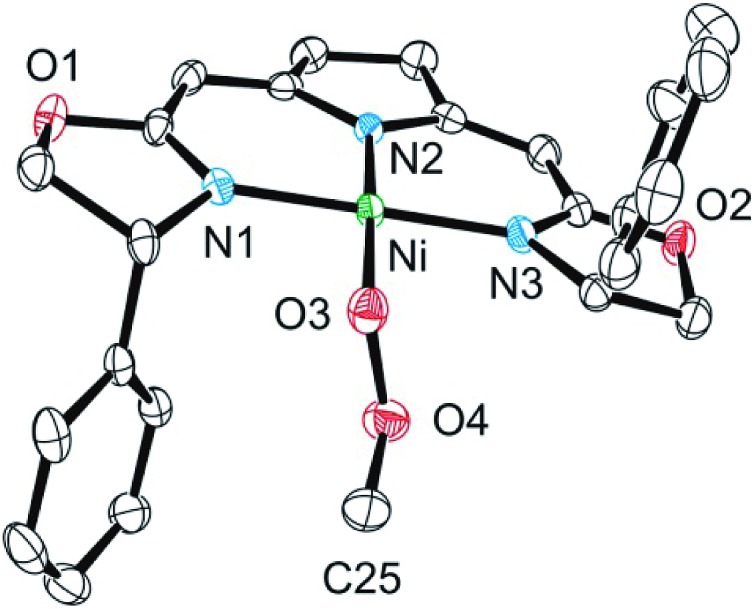
Molecular structure of the methylperoxo complex **15**. Hydrogen atoms were omitted for clarity. Selected bond lengths [Å] and angles [°]: Ni–O(3) 1.8497(16), Ni–N(1) 1.8927(19), Ni–N(2) 1.9246(19), Ni–N(3) 1.8948(19), O(3)–O(4) 1.513(2), O(4)–C(25) 1.398(3), O(3)–Ni–N(1) 87.57(8), O(3)–Ni–N(2) 173.91(8), O(3)–Ni–N(3) 87.96(8), N(1)–Ni–N(2) 92.43(8), N(1)–Ni–N(3) 174.55(8), N(3)–Ni–N(2) 92.35(8), O(4)–O(3)–Ni 102.51(11), C(25)–O(4)–O(3) 104.97(17), Ni–O(3)–O(4)–C(25) –170.27(15).

The methyl complex **14** slowly reacted with oxygen to give the methylperoxo complex **15**. In an analogous manner, oxygen has been shown previously to insert into the Zn–R and Pt–R bonds leading to the corresponding alkylperoxo complexes.^63^,^64^ The methylperoxo complex **15** was isolated and characterized. Similar to the hydroperoxo complex **5a** the apparent weakness of the O–O vibrational Raman band hampered the identification of this mode. However, suitable single crystals for X-ray diffraction were obtained (Fig. 5).

The molecular structure of **15** resembles to a large extent that of the corresponding hydroperoxo complex **5a**.^37^ Both complexes possess a square-planar coordination geometry with almost identical Ni–N and Ni–O bond lengths [Ni–O(3) 1.8497(16) (**15**), 1.8456(16) (**5a**); Ni–N(1) 1.8927(19) (**15**), 1.8962(18) (**5a**); Ni–N(2) 1.9246(19) (**15**), 1.9264(18) (**5a**); Ni–N(3) 1.8948(19) (**15**), 1.8889(19) (**5a**)]. However, the torsion angle Ni–O(3)–O(4)–C(25) of –170.27(15) of the peroxo ligand in **15** reflects the repulsive nature of the interaction of the methylperoxo ligand with the Ph-substituent of the oxazoline ring. The O–O bond length in **15** is elongated compared to **5a** [**15**: O(3)–O(4) 1.513(2), **5a**: 1.492(2)]. Both interatomic distances are rather large compared to Akita's ^*t*^Bu-peroxo species^65^ and other related complexes found in the literature.^66^–^69^

**Scheme 12:**
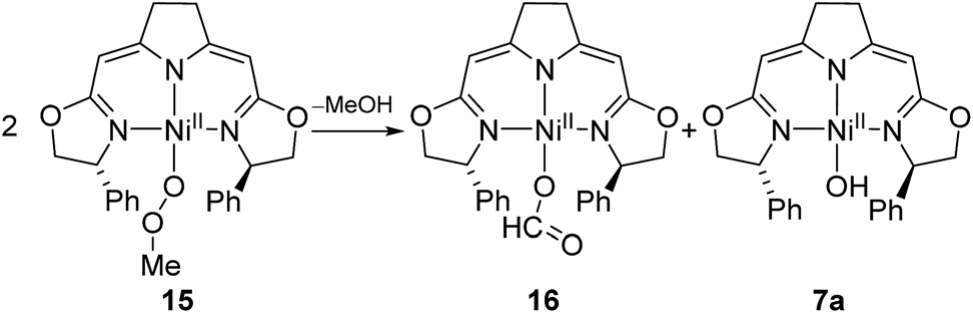
Thermal decomposition of the methylperoxo complex **9**.

The methylperoxo complex **15** was found to be fairly stable at room temperature but slowly converted to a mixture of near equal amounts of the formato and the hydroxo complexes **16** and **7a** along with half an equivalent of methanol (Scheme 12).

**Fig. 6:**
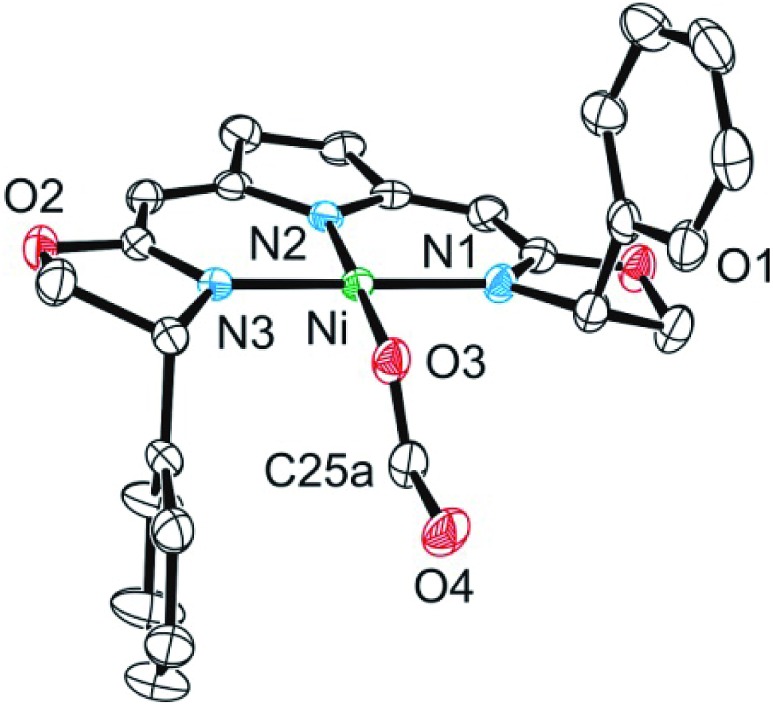
Molecular structure of formato complex **16**. Only the major of two disordered sites of the formato ligand is depicted. Hydrogen atoms were omitted for clarity. Selected bond lengths [Å] and angles [°]: Ni–O(3) 1.8850(15), Ni–N(1) 1.8934(17), Ni–N(2) 1.9001(17), Ni–N(3) 1.8894(17), O(3)–C(25A) 1.326(5), O(4)–C(25A) 1.228(5), O(3)–Ni–N(1) 87.35(7), O(3)–Ni–N(2) 178.36(7), O(3)–Ni–N(3) 87.33(7), N(1)–Ni–N(2) 93.00(7), N(3)–Ni–N(1) 173.73(8), N(3)–Ni–N(2) 92.41(8).

The formato complex **16** was also separately synthesized by the reaction of the hydroxo complex **7a** with formic acid, isolated and characterized by ^1^H, ^13^C NMR as well as IR spectroscopy. The molecular structure was established by X-ray diffraction and is depicted in Fig. 6. The C–O bond lengths of the formato ligand were found to be O(3)–C(25A) 1.326(5) Å and O(4)–C(25A) 1.228(5).

**Scheme 13:**
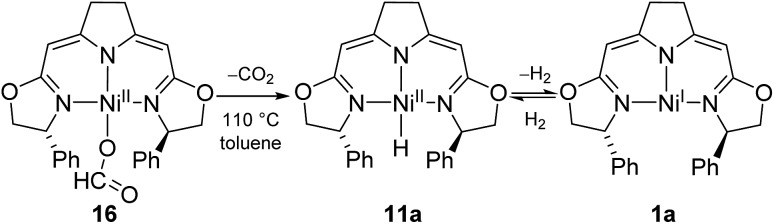
Thermal decomposition of formato complex **10**.

The formato complex **16** itself decomposed at elevated temperatures to CO_2_, dihydrogen as well as the nickel(i) species **1a** (Scheme 13). The formation of the latter is thought to occur *via* the hydrido complex **11a** which was detected in trace amounts (^1^H NMR) after the reaction was completed. Attempts to establish reaction conditions for the reverse reaction, the hydrogenation of CO_2_ in the presence of the nickel(i) complex, were unsuccessful.

## Conclusions

This study has shed further light onto the electronic structure and reactivity of the three- and four-coordinate nickel(i) complexes **1b** and **2b** bearing the iso-PyrrMeBox pincer ligand which partially models the structure of the hydrocorphin macrocycle in cofactor F430. However, this analogy is limited to the almost identical delocalized 10-electron-π-system involving three nitrogen donor atoms while the coordination of a fourth ligand was found occur only with the strong π-acceptor CO. This additional coordination significantly influences the electronic structure and is reflected in the way the *g* tensor of **1b**, which is highly anisotropic, is modified significantly towards an axial symmetry upon the occupation of the fourth coordination site in the CO-adduct **2b**, a spectroscopic characteristic reminiscent of the cofactor F430.

These low-valent T-shaped nickel complexes readily form peroxo species in the presence of dioxygen. While their formation and degradation may model aspects of the aerobic deactivation of methyl coenzyme M reductase (MCR), the dominance of dinuclear peroxo-species in the reaction pathways observed in this, limits the relevance of the model system **1b** for such processes involving the cofactor F430. However, the reaction patterns and structurally characterized intermediate and final oxidation products established in this study are expected to shed new light on the mechanisms operating in oxidations catalyzed by Ni complexes.

The most notable general principles appear to be the propensity of the Ni–X (X = O, C, H) bonds to be cleaved homolytically and the facile insertion of O_2_ into Ni–C bonds. The homolytic dissociation of Ni–O bonds in nickel peroxo species is believed to generate highly reactive radical intermediates which initiate the autoxidation process(es) observed. These are complex multistep reaction sequences which are only partially elucidated and define the challenges for future work in this field.

## Supplementary Material

Supplementary information

Crystal structure data
