# Supplementary material for: Electronic structure and reactivity of nickel(i) pincer complexes: their aerobic transformation to peroxo species and site selective C–H oxygenation
Source: Chem Sci. 2016 Feb 11;7(6):3533–42. doi: 10.1039/c5sc04644k (PMC6007179; doi:10.1039/c5sc04644k)
Supplement: Supplementary file 1 [file SC-007-C5SC04644K-s001.pdf]

Electronic Supplementary Material (ESI) for Chemical Science.
This journal is © The Royal Society of Chemistry 2016

Electronic Structure and Reactivity of Nickel(I) Pincer Complexes: Their Aerobic Transformation to Peroxo Species and Site Selective C-H Oxygenation

Christoph A. Rettenmeier, Hubert Wadepohl and Lutz H. Gade

Supporting Information

S1. Experimental Procedures

S1.1. General Procedures

S1.2 Preparation of Compounds

S1.3 Additional Experiments

S2. Crystallographic Data

S2.1. X-ray crystal structure determinations

S2.2. X-ray Crystal Structure of complex 10

S3. Computational Data

S1. Experimental Procedures

S1.1. General Procedures

All manipulations of air- and moisture-sensitive materials were carried out under an inert atmosphere of dry argon (Argon 5.0 purchased from Messer Group GmbH and dried over Granusic© phosphorpentoxide granulate) using standard Schlenk techniques or by working in a glove box. The solvents were dried over sodium (toluene), potassium (hexane) or sodium/potassium alloy (pentane, diethyl ether), distilled and degassed prior to their use.[1] Deuterated solvents were purchased from Deutero GmbH and or from Euriso-Top GmbH, dried over potassium (C_6D_6, toluene-d8, thf-d8), vacuum distilled, degassed and stored in teflon valve ampoules under argon. Hydrogen 5.0 (Messer Group GmbH), ethylene 3.0, carbon monoxide 4.8, oxygen 3.0, deuterium 2.7 (Air Liquide) and ^{18}O-labeled oxygen (99%, Sigma Aldrich) were used as purchased without further purification. The phenyl-, *iso*-propyl-, *tert*-butyl- and indandiyl-substituted 2,5-bis(oxazolinylmethyl)pyrrole protioligands (**Lig_{ph}H**, **Lig_{iPr}H**, **Lig_{tBu}H**, **Lig_{ind}H**)[2] as well as the complexes **Lig(iso)Ni (1a-d)**,[3] **Lig_{iPr}(iso)NiOAc**,[4] **Lig_{iPr}(iso)NiCl**,[3] **Lig_{Ph}(iso)NiBr**[3] and **Lig_{Ph}(iso)NiOH (7a)**[5] were synthesized according to literature procedures. All other reagents were obtained from commercial sources and were used as received unless explicitly stated otherwise.

Air-sensitive samples for NMR spectroscopy were prepared under argon in 5 mm Wilmad tubes equipped with J. Young Teflon valves. ^{1}H- and ^{13}C-NMR spectra were recorded on a Bruker Avance (200 MHz), a Bruker Avance II (400 MHz) and a Bruker Avance III (600 MHz, equipped with a CryoProbe™) NMR spectrometers and were referenced internally using the residual protio solvent (^{1}H) or solvent (^{13}C).[6] The appearance of the signals was described using the following abbreviations: s (singlet), d (doublet), dd (doublet of doublet), ddd (doublet of doublet of doublet), dt (doublet of triplet), t (triplet), q (quartet), m (multiplet), b (broad signal).

Continuous-wave X-band (ca. 9 GHz) EPR spectra were acquired using a Bruker Biospin Elexsys E500 EPR spectrometer fitted with a super high Q cavity. The magnetic field and the microwave frequency were calibrated with a Bruker ER 041XK Teslameter and a Bruker microwave frequency counter. The temperature of the sample was adjusted using a flow-through cryostat in conjunction with a Eurotherm (B-VT-2000) variable-temperature controller. EPR spectra simulations were carried out using the XSophe software (Bruker, version 1.1.4).

Resonance Raman samples were analyzed in solid state using a Horiba LABRAM n°2/781M spectroscope equipped with an Olympus BX40 microscope and a Linkam cooling device. The laser frequencies used were 632.817 nm and 473.08 nm.

Elemental analyses were recorded by the analytical service of the Heidelberg Chemistry Department using the vario EL and vario MIKRO cube analytical devices.

Mass spectra were acquired on Bruker ApexQe hybrid 9.4 T FT-IVR (HR-ESI, HR-DART) and JEOL JMS-700 magnetic sector (HR-FAB, HR-EI, LIFDI) spectrometers at the mass spectrometry facility of the Organic Department at the University of Heidelberg. Either 3-nitrobenzyl alcohol (NBA) or *o*-nitrophenyloctyl ether (NPOE) were used as matrix in the FAB-MS measurements.

X-Ray diffraction analysis was performed at the laboratory for structural analysis of the Inorganic Chemistry Department at the University of Heidelberg under the supervision of Prof. Dr. Wadepohl. An Agilent Technologies Supernova-E CCD (Cu-K_α or Mo-K_α X-radiation, microfocus tube,

multilayer mirror optics) and a Bruker AXS Smart 1000 CCD diffractometer (Mo (K_α) radiation, graphite monochromator, λ = 0.71073 Å) was used for data acquisition.

IR spectra were acquired on a Varian 3100 FT IR spectrometer (Excalibur series). Band intensities were classified using the following abbreviations: s = strong, m = medium, w = weak, b = broad.

UV/Vis spectra were recorded on a Varian Cary 5000 UV/VIS/NIR spectrometer.

S1.2. Preparation of Compounds

Preparation of Lig_{iPr}(iso)Ni(CO) (2b)

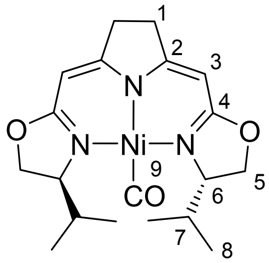

A suspension of Lig_{iPr}(iso)Ni (**1b**) (12.7 mg, 0.0339 mmol) in 0.2 ml hexane is subjected to 10 bar CO pressure, heated until a clear solution is obtained and finally kept at −30 °C to allow the product to crystallize. Subsequently, the solvent was decanted and completely removed by evaporation to give product **2b** as dark green crystalline solid in 72% (9.8 mg, 0.0243 mmol) yield. **^{1}H-NMR (toluene-d8, 600.130 MHz, 295 K):** δ (ppm) = 6.2, 4.2, 2.5, 1.9, −0.1 (Only the stated ^{1}H NMR signals and none of the ^{13}C NMR resonances could be detected in the corresponding NMR spectra due to paramagnetism). **Elemental analysis (%):** calculated for $C_{19}H_{26}N_3NiO_3$: C 56.61, H 6.50, N 10.42; found: C 56.47, H 6.66, N 10.49. **HR-MS (FAB+):** calculated for $C_{18}H_{26}N_3{}^{58}NiO_2$ $[M-CO]^+$: m/z = 374.1379, found: *m/z* = 374.1387. **EPR (X-band, 9.63284 GHz, toluene, 30 K):** g_x = 2.022 ($A_{N(oxazoline)}$ = 10.5 G, $A_{N(oxazoline)}$ = 10.5 G, $A_{N(central)}$ = 6 G), g_y = 2.111, g_z = 2.171. **IR (KBr):** 2954 (s), 1955 (s), 1582 (s), 1539 (m), 1512 (s), 1462 (w), 1424 (w), 1407 (w), 1342 (w), 1296 (m), 1259 (s), 1220 (s), 1059 (w), 1008 (s), 992 (s), 808 (w), 755 (m).

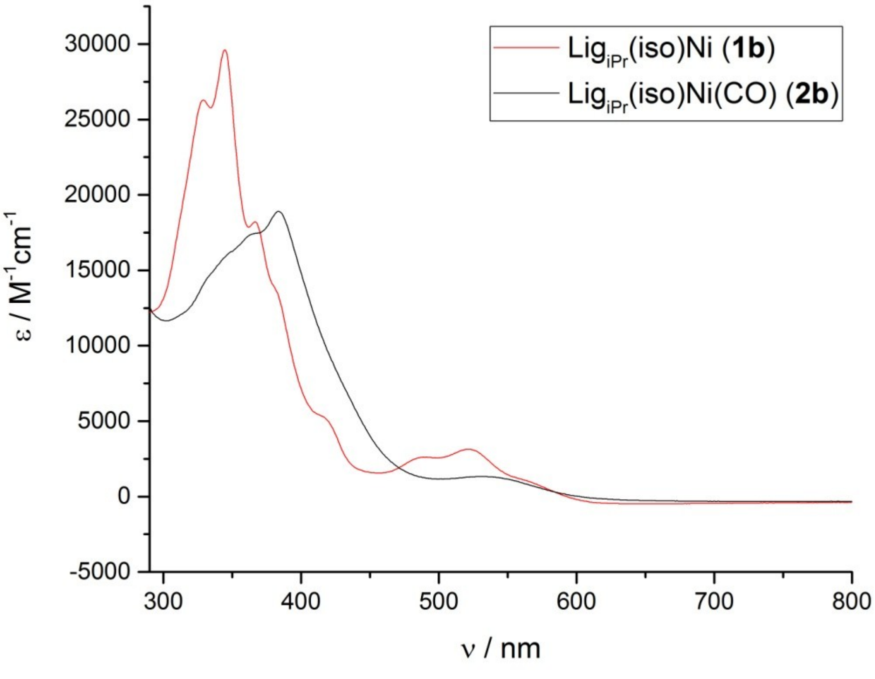

UV/Vis spectra of the THF solution of **1b** and **2b** which was formed after subjection of the former solution to 1 bar CO pressure.

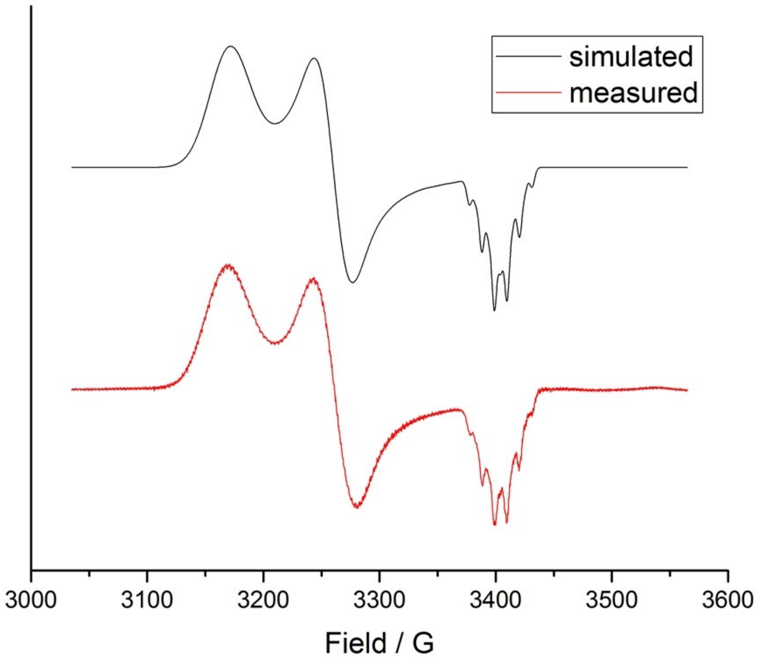

Measured (bottom) and simulated (top) X-band EPR spectra of the nickel(I) complex **2b** (9.63284 GHz, toluene, 30 K). Superhyperfine coupling to both the two equivalent N(oxazoline) atoms (C_2-symmetry) and the central N(central) atom is observed in x-component of the signal. Simulation details: XSophe (version 1.1.4); CW powder spectrum with H = $\beta B.g_e.S + S.A_{N(central)}.I + S.A_{2N(oxazoline)}.I$; grid: No. of portions: 100, No. of segments: 20; g_x = 2.022 ($a_{N(oxazoline)}$ = 10.5 G, $a_{N(oxazoline)}$ = 10.5 G, $a_{N(central)}$ = 6 G), g_y = 2.111, g_z = 2.171; line widths: 2.5 G (g_x), 15 G (g_y), 20 G (g_z).

Preparation of the µ-1,2-peroxo complex [Lig$_{tBu}$(iso)NiO]$_2$ (3c)

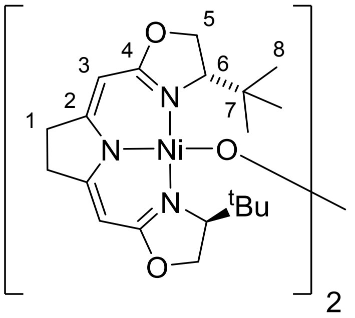

The solution of the nickel(I) complex **1c** in Et_2O was cooled to −78 °C, subjected to oxygen and held at low temperature for 15 min. After removal of the solvent at low temperature, the product was obtained as red solid. The obtained complexes are thermally instable and rapid decomposition was found to occur at temperatures above 0 °C. **^{1}H-NMR (toluene-d8, 399.890 MHz, 233 K):** δ (ppm) = 4.80 (s, 2 H, H^3), 4.51 (m, 2 H, H^6), 4.05 (m, 2 H, H^5), 3.86 (m, 2 H, $H^{5'}$), 1.96-1.45 (m, 22 H, $H^{1,1',8}$). **^{13}C NMR (toluene-d8, 100.552 MHz, 233 K):** δ (ppm) = 169.2 (C^2), 163.6 (C^4), 81.5 (C^3), 68.7 (C^5), 67.2 (C^6), 35.5 (C^7), 30.2 (C^1), 27.1 (C^8). **MS (Low Temperature FAB+)**: calculated for $C_{40}H_{60}N_6Ni_2O_6$: m/z = 836.3, found: m/z = 836.2. **Resonance Raman (solid, λ = 632 nm):** ν (cm^{-1}) = 779 cm^{-1}.

Preparation of the hydroperoxo complex Lig$_{iPr}$(iso)NiOOH (5b)

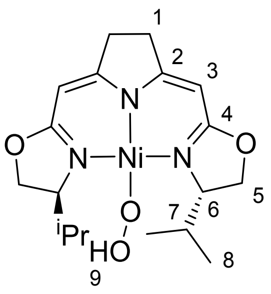

A solution of the ethyl complex **12** (28 mg, 0.069 mmol) in 0.6 ml toluene-d8 was subjected to 4 bar O_2 at −78 °C and analyzed by NMR spectroscopy at 233 K. Next to the hydroperoxo complex **5b** the formation of ethylene was observed. Compound **5b** was found to slowly convert to the 1,2-µ-peroxo complex **3b** at 233 K and attempts of isolation were unsuccessful. **^{1}H-NMR (toluene-d8, 600.130 MHz, 233 K):** δ (ppm) = 7.40 (b, 1 H, H^9), 4.83 (s, 2 H, H^3), 4.61 (m, 2 H, H^6), 3.93-3.72 (m, 4 H, $H^{5,5'}$), 2.92 (m, 2 H, H^7), 1.89-1.64 (m, 4 H, $H^{1,1'}$), 0.94-0.71 (m, 12 H, $H^{8,8'}$). **^{13}C-NMR (toluene-d8, 150.903**

MHz, 233 K): δ (ppm) = 169.4 (C^2), 163.0 (C^4), 81.2 (C^3), 67.1 (C^5), 65.2 (C^6), 32.5 (C^7), 30.0 (C^1), 19.1 (C^8), 14.7($C^{8'}$).
The hydroperoxo complex **5b** was also formed in the reaction of the corresponding chlorido complex **Lig$_{iPr}$(iso)NiCl** with H_2O_2 and in the reaction of **3b** with H_2O_2 at −78 °C.

Preparation of cyclic peroxo complexes 6/6'

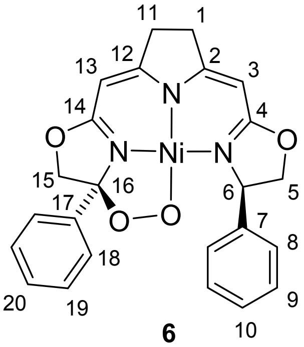

6

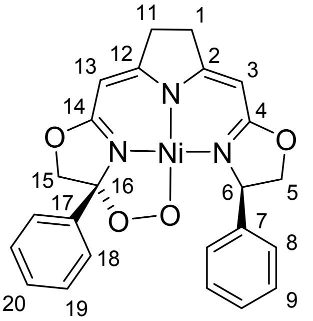

6'

Oxygen was bubbled through a solution of the nickel(I) complex **1a** (53 mg, 0.11 mmol) in 2 ml THF at −78 °C for 5 min. Subsequently, the reaction mixture was allowed to warm up to room temperature and stirred for 18 hours. The solution was flushed through a silica plug (washed with THF) and the solvents were removed under reduced pressure. After fractional precipitation from a toluene/pentane mixture, both products **6** and **6'** were obtained as a mixture in 42% yield (22 mg). Analytical data was reported previously.[5]

Preparation of the hydroxo complex Lig$_{iPr}$(iso)NiOH (7b)

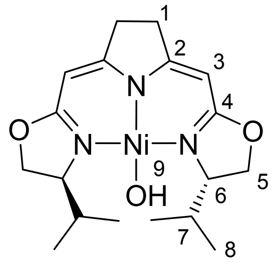

To a solution of Lig$_{iPr}$(iso)NiOAc (100 mg, 0.23 mmol) in 2 ml THF, NaOH (70 mg, 1.75 mmol) and 2 drops of H_2O were added and the reaction mixture was stirred for 3 h at room temperature. Subsequently, the solvents were removed under reduced pressure, toluene was added and the solution was filtered. After removal of the solvent, the product was obtained as yellow powder in 43% yield (38 mg). **^{1}H-NMR (toluene-d8, 600.130 MHz, 295 K):** δ (ppm) = 4.74 (s, 2 H, H^3), 3.99 (m, 2 H, H^6), 3.81 (m, 2 H, H^5), 3.72 (m, 2 H, $H^{5'}$), 2.67 (m, 2 H, H^7), 1.91 (m, 4 H, $H^{1,1'}$), 0.78 (m, 6 H, H^8), 0.75 (m, 6 H, $H^{8'}$), -5.96 (s, 1 H, H^9). **^{13}C NMR (toluene-d8, 150.903 MHz, 295 K):**
δ (ppm) = 169.8 (C^2), 163.1 (C^4), 81.2 (C^3), 66.9 (C^5), 65.2 (C^6), 31.9 (C^7), 30.2 (C^1), 19.2 (C^8), 14.6 ($C^{8'}$). **HR-MS (DART+)**: calculated for $C_{18}H_{27}N_3{}^{58}NiO_3$: m/z = 390.1328, found: *m/z* = 390.1357. **Elemental analysis (%):** calculated for $C_{18}H_{27}N_3NiO_2{\cdot}H_2O$: C 52.71, H 7.13, N 10.25; found: C 53.45, H 6.71, N 9.95.

Preparation of cyclic alkoxo complex 8

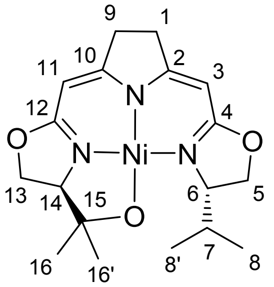

Oxygen was bubbled through a solution of the nickel(I) complex **1b** (50 mg, 0.13 mmol) in 3 ml diethyl ether at −78 °C for 10 min. Subsequently, the solvent was removed during which the temperature was held below −20 °C. After the addition of THF the reaction was allowed to warm up to room temperature under an atmosphere of argon and was stirred for 4 h. The solution was flushed through a silica plug (washed with THF) and the solvents were removed. The crude product was recrystallized from pentane at −40 °C to give an orange crystalline solid in 33% yield (17 mg). **^{1}H-NMR (C_6D_6, 600.130 MHz, 295 K):** δ (ppm) = 4.89 (s, 1 H, H^{11}), 4.88 (s, 1 H, H^3), 4.14 (ddd, ^3J = 9.2 Hz, ^3J = 3.2 Hz, ^3J = 3.2 Hz, 1 H, H^6), 4.05 (dd, ^3J = 12.1 Hz, ^3J = 8.8 Hz, 1 H, H^{14}), 3.94 (dd, ^3J = 8.6 Hz, ^3J = 3.3 Hz, 1 H, H^5), 3.89 (dd, ^3J = 8.7 Hz, ^3J = 8.6 Hz, 1 H, H^{13}), 3.65-3.58 (m, 2 H, $H^{5',13'}$), 3.19 (m, 1 H, H^7), 2.02-1.91 (m, 4 H, $H^{1,1',9,9'}$), 1.55 (s, 3 H, H^{16}), 1.17 (s, 3 H, $H^{16'}$),

1.00 (d, ^3J = 7.0 Hz, 3 H, H^8), 0.82 (d, ^3J = 7.1 Hz, 3 H, $H^{8'}$). **^{13}C-NMR (C_6D_6, 150.903 MHz, 295 K):** δ (ppm) = 170.8 (C^{10}), 168.9 (C^2), 163.0 (C^4), 161.9 (C^{12}), 81.1 (C^3), 80.2 (C^{11}), 80.1 (C^{14}), 75.1 (C^{15}), 72.1 (C^{13}), 67.6 (C^5), 66.0 (C^6), 31.2 (C^7), 30.5 (C^9) 30.0 (C^1), 28.0 (C^{16}), 26.6 ($C^{16'}$), 19.3 (C^8), 14.6 ($C^{8'}$). **HR-MS (ESI+)**: calculated for $C_{18}H_{25}N_3{}^{58}NiO_3$: m/z = 390.1328, found: *m/z* = 390.1320. **Elemental analysis (%):** calculated for $C_{18}H_{25}N_3NiO_3$: C 55.42, H 6.46, N 10.77, found: C 55.38, H 6.50, N 10.92.

Preparation of the cyclic peroxo complex 9

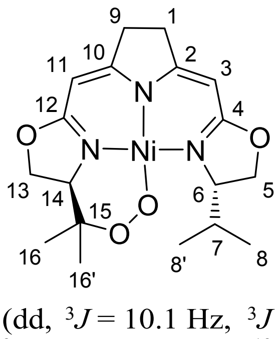

Oxygen was bubbled through a solution of the nickel(I) complex **1b** (98.0 mg, 0.26 mmol) in 2 ml toluene at −78 °C for 5 min. Subsequently, the reaction mixture was stirred at −20 °C for 10 min, allowed to warm up to room temperature and stirred for another hour. After the solvents were removed under reduced pressure, THF was added and the solution was flushed through a silica plug. After removal of the solvents, the product was recrystallized from pentane at −80 °C to give an orange crystalline solid in 48% yield (48.5 mg). **^{1}H-NMR (C_6D_6, 600.130 MHz, 295 K):** δ (ppm) = 4.91 (s, 1 H, H^{11}), 4.89 (s, 1 H, H^3), 4.48 (ddd, ^3J = 9.3 Hz, ^3J = 3.5 Hz, ^3J = 3.5 Hz, 1 H, H^6), 4.15 (dd, ^3J = 10.1 Hz, ^3J = 8.1 Hz, 1 H, H^{14}), 3.85-3.77 (m, 2 H, $H^{5,13}$), 3.64 (dd, ^3J = 8.3 Hz, ^3J = 8.3 Hz, 1 H, H^{13}), 3.58 (dd, ^3J = 9.0 Hz, ^3J = 8.9 Hz, 1 H, $H^{5'}$), 2.49 (dseptet, ^3J = 7.0 Hz, ^3J = 3.4 Hz, 1 H, H^7), 2.00-1.86 (m, 4 H, $H^{1,1',9,9'}$), 1.03 (s, 3 H, H^{16}), 0.99 (d, ^3J = 7.0 Hz, 3 H, H^8), 0.98 (s, 3 H, $H^{16'}$), 0.79 (d, ^3J = 7.0 Hz, 3 H, $H^{8'}$). **^{13}C-NMR (C_6D_6, 150.903 MHz, 295 K):** δ (ppm) = 170.5 (C^{10}), 169.4 (C^2), 163.7 (C^{12}), 163.3 (C^4), 81.6 (C^3), 80.1 (C^{11}), 78.5 (C^{15}), 78.5 (C^{14}), 69.1 (C^5), 67.4 (C^6), 64.7 (C^{13}), 32.5 (C^7), 30.5 (C^9) 29.9 (C^1), 25.9 ($C^{16'}$), 19.8 (C^{16}), 19.2 ($C^{8'}$), 15.0 (C^8). **HR-MS (FAB+)**: calculated for $C_{18}H_{26}N_3{}^{58}NiO_4$ $[M+H]^+$: m/z = 406.1277, found: *m/z* = 406.1274. **Elemental analysis (%):** calculated for $C_{18}H_{25}N_3NiO_4$: C 53.24, H 6.21, N 10.35 found: C 52.85, H 6.27, N 10.01.

Preparation of the oxazolinylcarboxylato complex 10

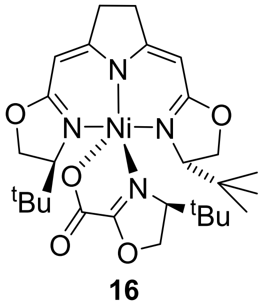

16

Oxygen was bubbled through a solution of the nickel(I) complex **1c** (30 mg, 0.074 mmol) in 2 ml toluene at −78 °C for 5 min. Subsequently, the reaction mixture was stirred at −20 °C for 10 min, allowed to warm up to room temperature and stirred for another 8 hours. After the solvents were removed under reduced pressure, the product was purified by fractional crystallization from a toluene/pentane mixture to give a brown crystalline solid in 28% yield (12 mg). **^{1}H-NMR (C_6D_6, 600.130 MHz, 295 K):** δ (ppm) = 33.42, 26.78, 18.82, 3.07, 0.86, −1.89, −11.34, −14.36, −26.33. The paramagnetic ^{13}C NMR signals could not be detected. **HR-MS (FAB+)**: calculated for $C_{28}H_{42}N_3{}^{58}NiO_5$ $[M]^+$: m/z = 572.2509, found: *m/z* = 572.2525. **Elemental analysis (%):** calculated for $C_{28}H_{42}N_4NiO_5$: C 58.66, H 7.38, N 9.77 found: C 58.60, H 7.11, N 9.63.

Preparation of the ethyl complex Lig_{iPr}(iso)NiEt (12)

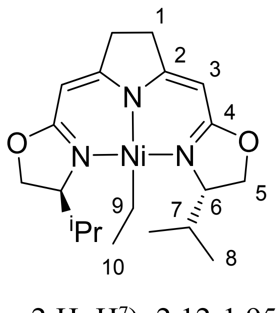

To a solution of the chlorido complex **Lig_{iPr}(iso)NiCl** (50 mg, 0.12 mmol) in 3 ml THF, EtMgCl (0.16 mmol, 1.3 eq) was added at −78 °C. After 5 min the cooling bath was removed and the reaction was stirred for another 20 min. After removal of the solvents, the residue was treated with a diethyl ether/pentane mixture (1/50) and the solution was filtrated. After removal of the solvents, the crude was recrystallized from hexane at −40 °C to give a yellow crystalline solid in 78% yield (38 mg). **^{1}H-NMR (C_6D_6, 600.130 MHz, 295 K):** δ (ppm) = 5.03 (s, 2 H, H^3), 3.99 (m, 2 H, H^6), 3.76 (dd, ^2J = 8.5 Hz, ^3J = 1.9 Hz, 2 H, H^5), 3.63 (dd, ^2J = 8.5 Hz, ^3J = 8.4 Hz, 2 H, $H^{5'}$), 2.40 (m, 2 H, H^7), 2.12-1.95 (m, 4 H, $H^{1,1'}$), 1.17 (dd, ^3J = 7.6 Hz, ^3J = 7.6 Hz, 3 H, H^{10}), 0.91-0.85 (m, 1 H, H^9), 0.80-0.74 (m, 1 H, $H^{9'}$) 0.72 (d, ^3J = 6.8 Hz, 6 H, H^8), 0.68 (d, 3 H, ^3J = 7.2 Hz, $H^{8'}$). **^{13}C-NMR (C_6D_6, 150.903 MHz, 295 K):** δ (ppm) = 170.7 (C^2), 164.3 (C^4), 80.8 (C^3), 68.5 (C^6), 66.0 (C^5), 33.3 (C^7), 30.9 (C^1), 18.9 ($C^{8'}$), 17.7(C^{10}), 14.9(C^8), 1.0(C^9). **HR-MS (LIFDI+)**: calculated for $C_{20}H_{31}N_3{}^{58}NiO_2$: *m/z* = 403.1; found *m/z* = 403.1. **Elemental**

analysis (%): calculated for $C_{20}H_{31}N_3NiO_2$: C 59.43, H 7.73, N 10.40; found: C 59.09, H 7.58, N 10.26.

Preparation of the hexenyl complex 13

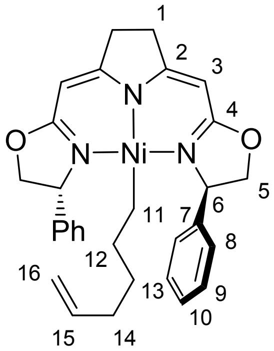

After the activation of Mg turnings (50 mg) using I_2, 6-bromohexene (70 mg, 0.43 mmol) dissolved in 5 ml THF was added and stirred for 6 h at room temperature. The reaction mixture was filtered and canulated to a solution of the nickel chlorido complex **Lig$_{Ph}$(iso)NiCl** (90 mg, 0.19 mmol) at 0 °C. After 20 min the solvents were removed and the residue was treated with pentane. The solution was stirred for 15 min and filtrated. After removal of the solvent, the product was obtained as yellow powder in 83% yield (83 mg). **^{1}H-NMR (C_6D_6, 600.130 MHz, 295 K):** δ = 7.21 (d, ^3J = 7.6 Hz, 4 H, H^8), 7.12 (m, 4 H, H^9), 7.02 (m, 2 H, H^{10}), 5.82 (m, 1 H, H^{15}), 5.17 (s, 2 H, H^3), 5.05 (m, 1 H, H^{16}), 4.98 (m, 1 H, $H^{16'}$), 4.91 (dd, ^3J = 2.0 Hz, ^3J = 8.4 Hz, 2 H, H^6), 3.76 (dd, ^2J = 8.4 Hz, ^3J = 8.0 Hz, 2 H, H^5), 3.65 (dd, ^3J = 2.4 Hz, ^2J = 8.0 Hz, 2 H, $H^{5'}$), 2,17 (m, 4 H, $H^{1,1'}$), 1.98 (m, 2 H, $H^{14,14'}$), 1.45 (m, 1 H, H^{12}), 1.17-1.30 (m, 3 H, $H^{12',13,13'}$), 0.70 (m, 1 H, H^{11}), 0.47 (m, 1 H, $H^{11'}$). **^{13}C-NMR (C_6D_6, 150.903 MHz, 295 K):** δ = 171.4 (C^2), 165.8 (C^4), 145.0 (C^7), 140.2 (C^{15}), 128.9 (C^9), 127.4 (C^{10}), 126.1 (C^8), 113.9 (C^{16}), 80.9 (C^3), 73.6 (C^5), 67.9 (C^6), 34.4 (C^{14}), 32.6 (C^{12}), 31.8 (C^{13}), 31.1 (C^1), 10.3 (C^{11}). **HR-MS (FAB+):** calculated for $C_{30}H_{33}N_3{}^{58}NiO_2$: m/z = 525.1926, found *m/z* = 525.1912. **Elemental analysis (%):** calculated for $C_{30}H_{33}N_3NiO_2$: C 68.46, H 6.32, N 7.98, found: C 68.62, H 6.10, N 8.29

Preparation of the methyl complex Lig$_{Ph}$(iso)NiMe (14)

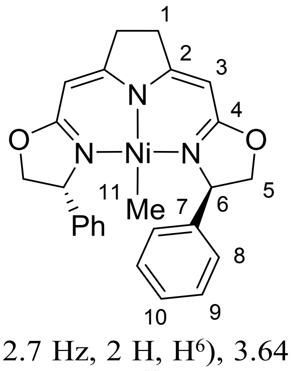

To a solution of the bromido complex **4a** (80 mg, 0.15 mmol) in 10 ml THF, MeMgBr (0.21 mmol, 1.4 eq) was added at −78 °C. After 20 min the cooling bath was removed and the reaction was stirred for another 5 min. After removal of the solvents, toluene was added (1 ml) and subsequently removed again. The residue was treated with a toluene/pentane mixture (1/20) and the solution was filtrated. After removal of the solvents, the crude was recrystallized from a toluene/pentane mixture (1/30) at −40 °C to give a yellow crystalline solid in 63% yield (44 mg). **^{1}H-NMR (C_6D_6, 600.130 MHz, 295 K):** δ (ppm) = 7.18 (d, ^{3}J = 7.6 Hz, 4 H, H^8), 7.12 (m, 4 H, H^9), 7.01 (tt, ^{3}J = 7.3 Hz, 1.2 Hz, 2 H, H^{10}), 5.16 (s, 2 H, H^3), 4.80 (dd, ^3J = 8.5 Hz, ^3J = 2.7 Hz, 2 H, H^6), 3.64 (dd, ^2J = 8.2 Hz, ^3J = 8.3 Hz, 2 H, H^5), 3.58 (dd, ^3J = 2.9 Hz, ^2J = 8.0 Hz, 2 H, $H^{5'}$), 2.20 (m, 4 H, $H^{1,1'}$),−0.07 (s, 3 H, H^{11}). **^{13}C-NMR (C_6D_6, 150.903 MHz, 295 K):** δ (ppm) = 171.3 (C^2), 165.4 (C^4), 145.1 (C^7), 128.9 (C^9), 127.4 (C^{10}), 126.2 (C^8), 80.8 (C^3), 73.6 (C^5), 67.3 (C^6), 31.0 (C^1), −7.9(C^{11}). **HR-MS (LIFDI+)**: calculated for $C_{25}H_{25}N_3{}^{58}NiO_2$ $[M-H]^+$: *m/z* = 457.1, found *m/z* = 456.9. **Elemental analysis (%):** calculated for $C_{26}H_{23}N_3NiO_2$: C 65.54, H 5.50, N 9.17, found: C 65.46, H 5.49, N 9.19.

Preparation of the methylperoxo complex Lig$_{Ph}$(iso)NiOOMe (15)

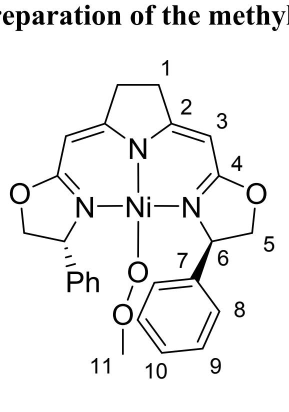

To a solution of the chlorido complex **Lig$_{Ph}$(iso)NiCl** (47 mg, 0.098 mmol) in 5 ml THF, MeMgBr (0.15 mmol, 1.5 eq) was added at −78 °C. After 5 min the cooling bath was removed and the reaction was stirred for another 20 min. After removal of the solvents, toluene was added (1 ml) and subsequently removed again. The residue was treated with a toluene/pentane mixture (1/20) and the solution was filtrated after centrifugation (1 h at 15 °C, 2000 rps). After removal of the solvents, the crude was dissolved in a toluene, pentane mixture (1/20), O_2 was added and the reaction

mixture was held at 4 °C for 24 hours and subsequently at −32 °C for 48 h to give a yellow crystalline solid. The solvent was decanted and the product was obtained in 71% yield (34 mg). **^{1}H-NMR (toluene-d8, 600.130 MHz, 295 K):** δ (ppm) = 7.38 (d, ^{3}J = 7.5 Hz, 4 H, H^8), 7.18 (dd, ^{3}J = 7.7 Hz, ^{3}J = 7.7 Hz, 4 H, H^9), 7.05 (t, ^{3}J = 7.4 Hz, 2 H, H^{10}), 5.31 (dd, ^3J = 8.5 Hz, ^3J = 2.0 Hz, 2 H, H^6), 4.88 (s, 2 H, H^3), 3.81 (dd, ^2J = 8.4 Hz, ^3J = 8.4 Hz, 2 H, H^5), 3.74 (dd, ^3J = 2.3 Hz, ^2J = 8.1 Hz, 2 H, H$^{5'}$), 3.07 (s, 3 H, H^{11}), 2.01-1.85 (m, 4 H, H$^{1,1'}$). **^{13}C-NMR (toluene-d8, 150.903 MHz, 295 K):** δ (ppm) = 170.3 (C^2), 164.5 (C^4), 145.7 (C^7), 128.5 (C^9), 127.1 (C^{10}), 126.5 (C^8), 81.3 (C^3), 74.8 (C^5), 64.0 (C^6), 62.2 (C^{11}), 30.2 (C^1). **HR-MS (DART+)**: calculated for $C_{24}H_{22}N_3{}^{58}NiO_2$ [M−OOMe]$^+$: *m/z* = 442.1065, found *m/z* = 442.10486. **Elemental analysis (%):** calculated for $C_{25}H_{25}N_3NiO_2{}^{18}O_2$: C 61.26, H 5.14, N 8.57, found: C 61.10, H 5.44, N 8.28.

Preparation of the formato complex Lig$_{Ph}$(iso)NiOOCH (16)

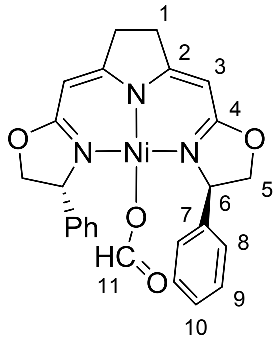

To a solution of the hydroxo complex **7a** (20 mg, 0.15 mmol) in 3 ml toluene a drop of HOOCH was added and stirred for 10 min. After removal of the volatiles, the crude was recystallized from a toluene/pentane mixture to give a red crystalline solid in 75% yield (16 mg). **^{1}H-NMR (C_6D_6, 600.130 MHz, 295 K):** δ (ppm) = 9.29 (s, 1 H, H^{11}), 7.27-7.13 (m, 8 H, H^{8,9}), 7.07 (t, ^3J = 6.9 Hz, 2 H, H^{10}), 5.09 (d, ^3J = 6.0 Hz, 2 H, H^6), 4.60 (s, 2 H, H^3), 3.18-3.52 (m, 4 H, H$^{5,5'}$), 1.80-1.62 (m, 4 H, H$^{1,1'}$). **^{13}C-NMR (C_6D_6, 150.903 MHz, 295 K):** δ (ppm) = 172.5 (C^2), 167.4 (C^4), 161.6 (C^{11}), 144.5 (C^7), 129.0 (C^9), 127.8 (C^{10}), 126.3 (C^8), 83.2 (C^3), 76.7 (C^5), 68.6 (C^6), 35.5 (C^1). **HR-MS (DART+)**: calculated for $C_{24}H_{22}N_3{}^{58}NiO_3$ [M−HCO]$^+$: *m/z* = 458.1009 found *m/z* = 458.1007. **Elemental analysis (%):** calculated for $C_{25}H_{23}N_3NiO_4$: C 61.51, H 4.75, N 8.61, found: C 61.71, H 4.79, N 8.55. **IR (KBr):** 2968 (w), 2909 (w), 2809 (m), 2704(s), 1963 (w), 1888 (w), 1816 (w), 1755 (w), 1647 (s), 1641 (s), 1612 (s), 1538 (s), 1452 (m), 1341 (m), 1252 (s), 1221 (s), 1069 (m), 1021 (s), 771 (m), 735(s).

S1.3. Additional Experiments

Comparison of the decomposition rates of 3b under argon and 8 bar oxygen pressure

In both experiments 0.4 ml of a stock solution of 20.2 mg of the nickel(I) complex **1b** in 1.6 ml THF were used. The samples were subjected to 8 bar O_2 at −78 °C and held at low temperature for 15 min. In first experiment the sample was allowed to warm to room temperature (295 K) under 8 bar oxygen pressure and the reaction was monitored by ^{1}H NMR spectroscopy. The integral of the ^{1}H NMR signal at 5.71 ppm (H^6, THF, 295 K)[5] was used to determine the concentration of **3b** throughout the course of the reaction. In the second experiment the sample was degassed at low temperature prior to allowing it to warm to room temperature. No significant difference in the decomposition rate in both experiments was observed (see Figure 4).

Determination of the aerobic decomposition rates of 3a and 3b

The corresponding solutions of the nickel(I) complexes (11.5 mg (1a) and 9.7 mg (1b) and 1,4-dimethoxybenzene in 0.5 ml toluene-d8) were subjected to 8 bar O_2 at −78 °C und held for 15 min at low temperature. Subsequently, the samples were allowed to warm to 283 K and the decomposition reaction was monitored by ^{1}H NMR spectroscopy (integrals of the ^{1}H NMR signals at 5.98 ppm (H^6)[5] for **3b** and 6.37 ppm (H^6)[5] for **3a** in toluene at 285 K were used, respectively).

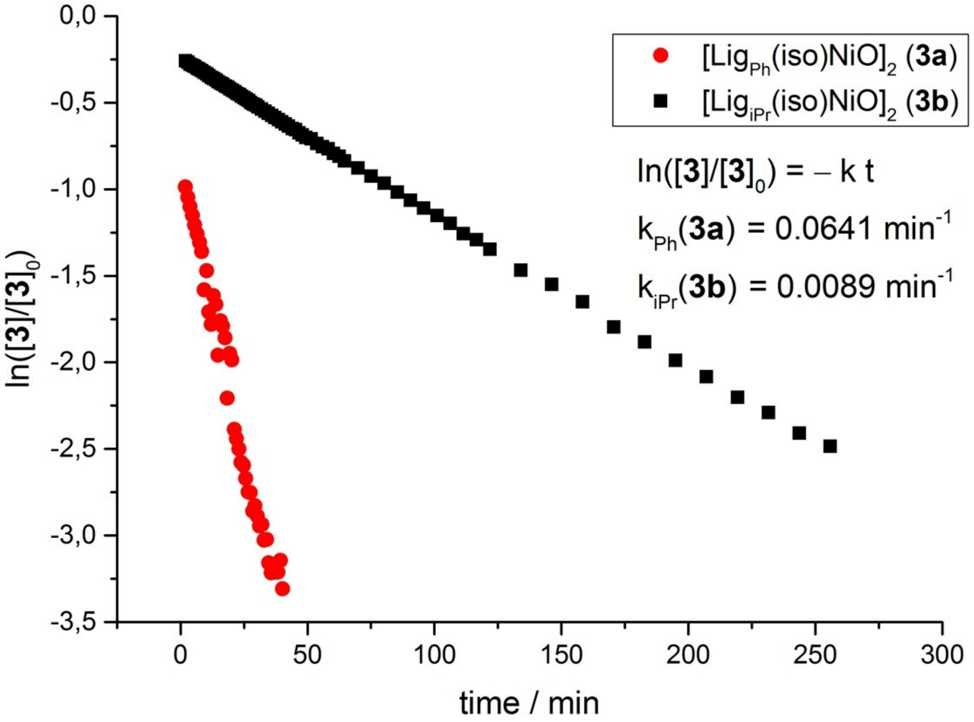

Course of the thermal decomposition of Ph- and ^{i}Pr -substituted µ-1,2-peroxo complexes **3a,b** in the presence of oxygen (5 bar) in toluene monitored by ^{1}H NMR spectroscopy.

Labeling Experiments

Reaction of the hydroxo complex 7a with a mixture of $^{16}O_2$ and $^{18}O_2$

A solution of the hydroxo complex **7a** (6 mg,) in 0.4 ml THF was subjected to 4 bar of a mixture of $^{16}O_2$ and $^{18}O_2$ and held at room temperature for a week. The sample was analyzed by HR-MS(ESI+). The molecular ion peaks of both, the twofold ^{16}O and ^{18}O labeled cyclic peroxo complexes **6**/**6'**, but not of the scrambled products **6[$^{16}O^{18}O$]/6'[$^{16}O^{18}O$]** were observed.

Decomposition of 3b in the presence of a mixture of $^{16}O_2$ and $^{18}O_2$

A diethyl ether solution of **1b** was subjected to a mixture of $^{16}O_2$/$^{18}O_2$ at −78 °C and stirred at −10 °C for 20 h. Subsequently, the reaction was allowed to warm to room temperature and analyzed by HR-MS (ESI+). The molecular ion peaks of both, the twofold ^{16}O and ^{18}O labeled cyclic peroxo complexes **6**/**6[^{18}O]**, but not of the scrambled product **6[$^{16}O^{18}O$]** were observed

Reaction of the cyclic alkylperoxo complex 9 with the hydrido complex 11b

0.4 ml of a stock solution (16 mg in 0.8 ml toluene-d8 and 1,4-dimethoxybenzene as internal standard) of the nickel(I) complex **1b** were subjected to 8 bar oxygen at −78 °C for 10 min and subsequently allowed to warm to room temperature and held at room temperature for about 20 min until the reaction to the hydroxo complex **7b** and the cyclic peroxo complex **9** had completed (NMR). The solution was degassed and combined with a second sample of the *in situ* generated nickel hydrido complex **11b** (0.2 ml of the stock solution was subjected to 8 bar H_2) at room temperature. NMR analysis of the

sample showed that after 5 min about 50% of **9** had converted to **8** and after 4 h the transformation was complete.

Reaction of the hydroperoxo complex 5a with the hydrido complex 11a

The solution of the hydroperoxo complex **5a** (6,4 mg) in 0.5 ml THF-d8 and the solution 0.2 ml THF-d8 of the in situ generated hydrido complex **11a** (6.2 mg of **1a**, 10 bar H_2) were combined at room temperature and kept under 10 bar H_2 pressure. NMR analysis after less than 10 min showed a near complete conversion to the hydroxo complex **7a**.

Reaction of the nickel(I) complex 1b with N_2O

A solution of the nickel(I) complex **1b** (8 mg) in 0.4 ml toluene-d8 was cooled to −78 °C and subjected to N_2O. After about 10 min, the solution was analyzed by NMR spectroscopy at −30 °C. The formation of the hydrido complex **11b** (roughly 0.3 eq.) as well as **7b**, **9** and other species was observed.

Reaction of alkyl complexes 12 and 13 with oxygen

The solution of the ethyl complex **12** (28 mg) in 0.4 ml toluene-d8 was subjected to 5 bar O_2 at −78 °C in an NMR tube. After approximately 15 min the sample was analyzed by NMR spectroscopy at 233 K which showed that a clean formation to **5b** and ethylene had occurred. The latter slowly converted into the 1,2-μ-peroxo complex **3b**.

The solution of the hexenyl complex **13** (20 mg) in 0.4 ml toluene-d8 was subjected to 5 bar O_2 at 0 °C in an NMR tube. After approximately 5 min the sample was analyzed by NMR spectroscopy at 0 C which showed that a clean formation to **5b** and 1,5-hexadiene had occurred.

Thermal decomposition of the methylperoxo complex 15

The solution of the methylperoxo complex **15** (5 mg) in 0.4 ml toluene-d8 was held at room temperature for a week and was subsequently analyzed by NMR spectroscopy. The formation of the formato complex **16**, the hydroxo complex **7a** and methanol was observed.

Thermal decomposition of the formato complex 16

The solution of the formato complex **16** (5 mg) in 0.4 ml toluene-d8 was held at 110 C for 2 days. The subsequently NMR spectroscopic analysis at room temperature revealed that the nickel(I) complex **1a** and traces of the hydrido complex **11a** and H_2.

S2. Crystallographic Data

S2.1. X-ray crystal structure determinations

Crystal data and details of the structure determinations are compiled in Table S1. Full shells of intensity data were collected at low temperature with a Bruker AXS Smart 1000 CCD diffractometer (Mo-K_a radiation, sealed X-ray tube, graphite monochromator; compounds **8** and **10**) or an Agilent Technologies Supernova-E CCD diffractometer (Mo- or Cu-K_a radiation, microfocus X-ray tube, multilayer mirror optics; all other compounds). Data were corrected for air and detector absorption, Lorentz and polarization effects;[7,8] absorption by the crystal was treated numerically (Gaussian grid)[8,9] or with a semiempirical multiscan method.[10-13] The structures were solved by the heavy atom method combined with structure expansion by direct methods applied to difference structure factors[14,15] or by the charge flip procedure[16,17] and refined by full-matrix least squares methods based on F^2 against all unique reflections.[18-20] All non-hydrogen atoms were given anisotropic displacement parameters. Hydrogen atoms were generally input at calculated positions and refined with a riding model. When justified by the quality of the data the positions of some hydrogen atoms were taken from difference Fourier syntheses and refined. The disordered formato ligand in complex **16** was subjected to suitable geometry and adp restraints.

CCDC 1439102-1439106 contains the supplementary crystallographic data for this paper. These data can be obtained free of charge from The Cambridge Crystallographic Data Centre via www.ccdc.cam.ac.uk/data_request/cif.

S2.2. X-ray Crystal Structure of complex 10

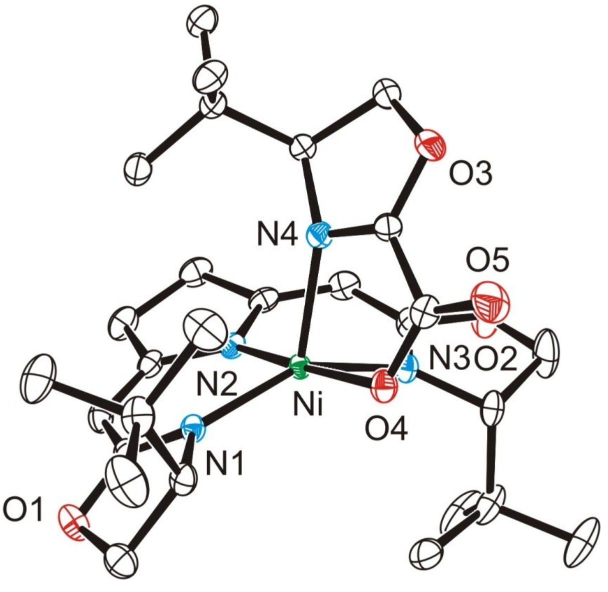

Molecular structure of complex **10**. Hydrogen atoms were omitted for clarity. Selected bond lengths [Å] and angles [°]: Ni-O(4) 2.0704(17), Ni-N(1) 2.0145(17), Ni-N(2) 2.0061(19), Ni-N(3) 2.0310(19), Ni-N(4) 2.052(2), O(4)-Ni-N(1) 92.90(7), O(4)-Ni-N(2) 175.82(6), O(4)-Ni-N(3) 88.30(6), N(1)-Ni-N(2) 91.27(7), N(1)-Ni-N(3) 151.74(7), N(3)-Ni-N(2) 87.87(7), O(4)-Ni-N(4) 80.43(7).

Table S1. Details of the crystal structure determinations of **8**, **9**, **10**, **15** and **16**.

	**8**	**9**	**10**	**15**	**16**
formula	$C_{18}H_{25}N_3NiO_3$	$C_{18}H_{25}N_3NiO_4$	$C_{28}H_{42}N_4NiO_5$	$C_{25}H_{25}N_3NiO_4$	$C_{25}H_{23}N_3NiO_4$
crystal system	monoclinic	orthorhombic	orthorhombic	orthorhombic	orthorhombic
space group	$P\ 2_1$	$P\ 2_12_12_1$	$P\ 2_12_12_1$	$P\ 2_12_12_1$	$P\ 2_12_12_1$
a /Å	11.854(6)	6.25709(10)	11.065(5)	6.41635(7)	10.14890(6)
b /Å	6.070(3)	16.6261(3)	13.046(6)	10.31527(11)	12.08020(7)
c /Å	12.641(6)	17.4692(2)	20.098(9)	32.9233(5)	17.62895(14)
β /°	104.097(6)				
V /Å^3	882.2(7)	1817.34(5)	2901(2)	2179.07(5)	2161.32(2)
Z	2	4	4	4	4
M_r	390.12	406.12	573.36	490.19	488.17
F_{000}	412	856	1224	1024	1016
d_c /Mg□m^{-3}	1.469	1.484	1.313	1.494	1.500
μ /mm^{-1}	1.122	1.096	0.711	1.608	1.621
max., min. transmission factors	0.7464, 0.6481	0.904, 0.587	0.7464, 0.7008	0.946, 0.801	1.0000, 0.8617
X-radiation, λ /Å	Mo-K□, 0.71073	Mo-K□, 0.71073	Mo-K□, 0.71073	Cu-K□, 1.54184	Cu-K□, 1.54184
data collect. temperat. /K	100(2)	110(1)	100(1)	120(1)	120(1)
θ range /°	1.7 to 30.5	3.4 to 32.5	1.9 to 32.5	4.5 to 70.9	4.4 to 70.8
index ranges h,k,l	-16 ... 16, -8 ... 8, -17 ... 17	-9 ... 9, -25 ... 25, -26 ... 26	-16 ... 16, -19 ... 19, -30 ... 29	-7 ... 7, -12 ... 12, -40 ... 38	-12 ... 12, -14 ... 14, -21 ... 19
reflections measured	21092	155621	75552	73508	109930
unique [R_{int}]	5365 [0.0512]	6454 [0.0945]	101108[0.0443]	4182 [0.0438]	4140 [0.045
observed [$I \geq 2\sigma(I)$]	4646	5596	8894	4156	4086
data / restraints /parameters	5365 / 1 / 273	6454 / 0 / 239	10108 / 0 / 352	4182 / 0 / 366	4140 / 27 / 317
GooF on F^2	0.977	1.117	1.042	1.094	1.065
R indices [$F>4\sigma(F)$] $R(F)$, $wR(F^2)$	0.0345, 0.0631	0.0451, 0.1250	0.0354, 0.0832	0.0228, 0.0575	0.0205, 0.0526
R indices (all data) $R(F)$, $wR(F^2)$	0.0463, 0.0662	0.0577, 0.1310	0.0448, 0.0879	0.0229, 0.0576	0.0208, 0.0528
absolute structure parameter	0.013(7)	-0.004(5)	0.004(4)	0.004(6)	-0.004(5)
largest residual peaks /e Å^{-3}	0.452, -0.345	0.922, -0.679	0.708, -0.244	0.394, -0.229	0.171, -0.190

S3. Computational Data

DFT calculations were performed using the Gaussian 09, Revision D.01 software package[21] on the bwforcluster JUSTUS. The geometry optimization and the harmonic frequency analysis were carried out on the restricted/restricted open shell B3LYP/6-311G(d,p) [22-33] level of theory using the "tight" convergence criteria for SCF calculations.

Cartesian coordinates of DFT optimized structures

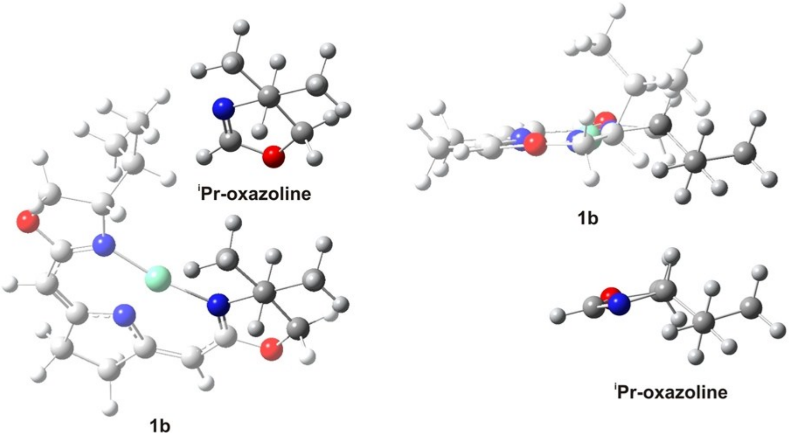

Comparison of the DFT optimized structures of **1b** and free ^{i}Pr-oxazoline reveals that the ^{i}Pr-substituent of **1b** adopts the same conformation as metal-free ^{i}Pr-oxazoline itself giving an explanation for the orientation of the methyl C-H group of the substituent in **1b** that does not involve a Ni···H interaction at all.

Complex **1b**

E = -2524.89617101 a.u.; G = -2524.528622 a.u.

Ni	0.09766100	0.03729300	-0.16667500
O	-3.92936500	0.45394800	-0.67026100
O	4.03905800	0.66699500	0.66045200
N	-1.77274900	-0.17797800	-0.45987500
N	1.97260800	-0.09077300	0.15318100
N	0.01884200	1.99648400	-0.04477300
C	-2.53311900	-1.43960900	-0.43904700
H	-2.14121400	-2.10653800	-1.21414200
C	-3.96104200	-0.98176000	-0.80663200
H	-4.22091500	-1.22194200	-1.84095700
H	-4.73969900	-1.36534200	-0.14635600
C	-2.62065300	0.81678600	-0.53706600
C	-2.35046200	2.20807000	-0.47401200
H	-3.19632900	2.86934600	-0.60089700
C	-1.10381000	2.73408300	-0.23368900
C	-0.79998700	4.22146600	-0.14681500
H	-1.42726100	4.70845500	0.60363600
H	-1.00804400	4.70819700	-1.10369500
C	0.69969900	4.25500000	0.21144900
H	1.29408300	4.83058100	-0.50210900
H	0.87990400	4.68575800	1.20032100
C	1.09401100	2.78652600	0.19448400
C	2.37065100	2.32242300	0.40391100
H	3.17022000	3.02449700	0.59571200
C	2.73125900	0.95089400	0.38756600
C	4.22299500	-0.74077600	0.42350600
H	4.79962900	-1.15358300	1.25101300
H	4.78966800	-0.85980800	-0.50662900

C	2.79245600	-1.30820600	0.30467100
H	2.49912500	-1.80878600	1.23901900
C	2.62068900	-2.31308100	-0.84902300
H	2.93174300	-1.79703500	-1.76677200
C	1.16106500	-2.74321900	-1.01940400
H	0.52349000	-1.87672700	-1.23375900
H	0.78610200	-3.23236800	-0.11414600
H	1.05436300	-3.44472200	-1.85137600
C	3.52630100	-3.53620700	-0.64260800
H	3.41539200	-4.24347000	-1.46863800
H	3.26554100	-4.06339600	0.28153900
H	4.58406400	-3.26425200	-0.58536100
C	-2.37689700	-2.16429400	0.91895000
H	-1.29337400	-2.27527700	1.05101800
C	-2.89478500	-1.34204700	2.10461800
H	-3.97677600	-1.18434200	2.05216700
H	-2.40913200	-0.36525300	2.14500800
H	-2.68642600	-1.85976000	3.04501100
C	-3.00211500	-3.56420700	0.87658700
H	-2.60126700	-4.15883100	0.04983700
H	-4.08979000	-3.51580700	0.75670400
H	-2.80356800	-4.10652800	1.80481100

^{i}Pr-oxazoline
E = -365.32224676 a.u.; G = -365.189455 a.u.

O	-2.28107700	0.54485000	-0.18425600
N	-0.87685500	-1.20256400	0.17819400
C	-1.00220300	1.18108100	0.09067600
H	-1.15051700	1.89905000	0.89687600
H	-0.69501100	1.71063600	-0.81521600
C	-0.05725900	0.00145300	0.44994000
H	0.17799800	0.00456500	1.52230100
C	1.27072700	-0.01476800	-0.32849100
H	1.01331300	-0.01228900	-1.39639400
C	2.07065400	-1.28795800	-0.03114300
H	1.47658300	-2.17779700	-0.24010000
H	2.36859200	-1.32200000	1.02269400
H	2.98172800	-1.32199800	-0.63530200
C	2.09978400	1.24139000	-0.02408900
H	3.03422700	1.23445600	-0.59129600
H	2.35908900	1.28724000	1.03927800
H	1.56975300	2.16389100	-0.27921600
C	-2.02714800	-0.78695800	-0.14115700
H	-2.87648400	-1.41203400	-0.39134500

Complex **2b**

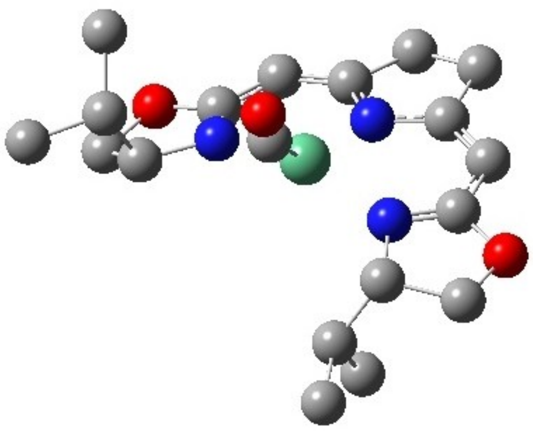

E = -2638.26341592 a.u.; G = -2637.894045 a.u.

Ni	-0.02990600	-0.06111600	-0.40087300
O	-4.22270400	0.28388300	-0.42001400
O	3.62149000	0.94461200	1.43058400
N	-2.04294800	-0.27829200	-0.32542200
N	1.84135700	0.02989700	0.38600100
N	-0.27991000	1.93867400	-0.06540800
C	-2.74744500	-1.56252200	-0.20898700
H	-2.43979400	-2.20841600	-1.03962400
C	-4.23442800	-1.16120600	-0.36966100
H	-4.67620200	-1.52853500	-1.29802300
H	-4.86694300	-1.46040500	0.46741000
C	-2.92084200	0.67598400	-0.41389000
C	-2.67256500	2.07258600	-0.49355400
H	-3.52847800	2.71334400	-0.65130900
C	-1.43238600	2.62536700	-0.32034100
C	-1.18265600	4.12282800	-0.34875700
H	-1.94858600	4.67399100	0.19930200
H	-1.20579200	4.47544100	-1.38480700
C	0.22129700	4.24583900	0.26053600
H	0.89377500	4.88625200	-0.31347900
H	0.18762100	4.64218500	1.27978000
C	0.70931100	2.80635600	0.28611200
C	1.96715000	2.45777600	0.69927100
H	2.64964000	3.22603000	1.03484200
C	2.42072900	1.11698200	0.81101500
C	3.79294700	-0.47692800	1.60772900
H	3.60087800	-0.71442100	2.65790100
H	4.82725300	-0.72322000	1.36970300
C	2.74960300	-1.10615800	0.65975500
H	2.19166900	-1.89161000	1.17768100
C	3.34363500	-1.72607000	-0.62891200
H	2.48871600	-2.05280700	-1.22688600
C	4.17179500	-2.97637000	-0.29894000
H	3.59127700	-3.69991500	0.28190300
H	5.07167400	-2.73127900	0.27493100
H	4.49920400	-3.47329700	-1.21583100
C	4.13931300	-0.72282300	-1.47250700
H	4.47427000	-1.19123700	-2.40197300
H	5.03026500	-0.36157900	-0.94904000
H	3.52796500	0.14244200	-1.73611200
C	-2.38306200	-2.30028500	1.10081800
H	-1.29698600	-2.44528400	1.04763200

C	-2.67453600	-1.47170000	2.35715600
H	-3.74487300	-1.27727000	2.48158800
H	-2.15730800	-0.51099000	2.31780200
H	-2.33547700	-2.00255600	3.25101200
C	-3.04272000	-3.68415000	1.15735200
H	-2.80668800	-4.27742400	0.26859000
H	-4.13295900	-3.60921000	1.23037700
H	-2.69832700	-4.24263400	2.03170200
C	0.17428300	-1.29428500	-1.70927200
O	0.17815700	-1.76920000	-2.75386400

1. W. L. F. Armarego and C. L. L. Chai, *Purification of Laboratory Chemicals*, Elsevier/Butterworth-Heinemann, 2009.
2. F. Konrad, J. Lloret Fillol, H. Wadepohl and L. H. Gade, *Inorg. Chem.*, 2009, **48**, 8523.
3. C. Rettenmeier, H. Wadepohl and L. H. Gade, *Chem. Eur. J.*, 2014, **20**, 9657.
4. F. Konrad, J. Lloret Fillol, C. Rettenmeier, H. Wadepohl and L. H. Gade, *Eur. J. Inorg. Chem.*, 2009, **2009**, 4950.
5. C. A. Rettenmeier, H. Wadepohl and L. H. Gade, *Angew. Chem. Int. Ed.*, 2015, **54**, 4880.
6. G. R. Fulmer, A. J. M. Miller, N. H. Sherden, H. E. Gottlieb, A. Nudelman, B. M. Stoltz, J. E. Bercaw and K. I. Goldberg, *Organometallics*, 2010, **29**, 2176.
7. *SAINT*, Bruker AXS GmbH, Karlsruhe, Germany, 1997.
8. *CrysAlisPro*, Agilent Technologies UK Ltd., Oxford, UK, 2011-2014 and Rigaku Oxford Diffraction, Rigaku Polska Sp.z o.o., Wrocław, Poland 2015.
9. W. R. Busing, H. A. Levy, *Acta Cryst.* 1957, **10**, 180.
10. G. M. Sheldrick, *SADAB*, Bruker AXS GmbH, Karlsruhe, Germany, 2004-2014.
11. L. Krause, R. Herbst-Irmer, G. M. Sheldrick, D. Stalke, *J. Appl. Cryst.* 2015, **48**, 3.
12. *SCALE3 ABSPACK, CrysAlisPro*, Agilent Technologies UK Ltd., Oxford, UK 2011-2014 and Rigaku Oxford Diffraction, Rigaku Polska Sp.z o.o., Wrocław, Poland 2015.
13. R. H. Blessing, *Acta Cryst.* 1995, **A51**, 33.
14. P. T. Beurskens, G. Beurskens, R. de Gelder, J. M. M. Smits, S. Garcia-Granda, R. O. Gould, *DIRDIF-2008*, Radboud University Nijmegen, The Netherlands, 2008.
15. P. T. Beurskens, in: G. M. Sheldrick, C. Krüger, R. Goddard (eds.), *Crystallographic Computing 3*, Clarendon Press, Oxford, UK, 1985, p. 216.
16. L. Palatinus, *SUPERFLIP*, EPF Lausanne, Switzerland, 2007-2014 and Fyzikální ústav AV ČR, v. v. i., Prague, Czech Republic, 2007-2014.
17. L. Palatinus and G. Chapuis, *J. Appl. Crystallogr.*, 2007, **40**, 786.
18. G. M. Sheldrick, *SHELXL-20xx*, University of Göttingen and Bruker AXS GmbH Karlsruhe, Germany, 2012-2014.
19. G. M. Sheldrick, *Acta Cryst.* 2008, **A64**, 112.
20. G. M. Sheldrick, *Acta Cryst.* 2015, **C71**, 3.
21. M. J. Frisch, G. W. Trucks, H. B. Schlegel, G. E. Scuseria, M. A. Robb, J. R. Cheeseman, G. Scalmani, V. Barone, B. Mennucci, G. A. Petersson, H. Nakatsuji, M. Caricato, X. Li, H. P. Hratchian, A. F. Izmaylov, J. Bloino, G. Zheng, J. L. Sonnenberg, M. Hada, M. Ehara, K. Toyota, R. Fukuda, J. Hasegawa, M. Ishida, T. Nakajima, Y. Honda, O. Kitao, H. Nakai, T. Vreven, J. J. A. Montgomery, J. E. Peralta, F. Ogliaro, M. Bearpark, J. J. Heyd, E. Brothers, K. N. Kudin, V. N. Staroverov, T. Keith, R. Kobayashi, J. Normand, K. Raghavachari, A. Rendell, J. C. Burant, S. S. Iyengar, J. Tomasi, M. Cossi, N. Rega, J. M. Millam, M. Klene, J. E. Knox, J. B. Cross, V. Bakken, C. Adamo, J. Jaramillo, R. Gomperts, R. E. Stratmann, O. Yazyev, A. J. Austin, R. Cammi, C. Pomelli, J. W. Ochterski, R. L. Martin, K. Morokuma, V. G. Zakrzewski, G. A. Voth, P. Salvador, J. J. Dannenberg, S. Dapprich, A. D. Daniels, O. Farkas, J. B. Foresman, J. V. Ortiz, J. Cioslowski and D. J. Fox, *Journal*, 2013, **Gaussian 09, Revision D.01**.
22. A. J. H. Wachters, *J. Chem. Phys.*, 1970, **52**, 1033.

23. P. J. Hay, *J. Chem. Phys.*, 1977, **66**, 4377.
24. R. Krishnan, J. S. Binkley, R. Seeger and J. A. Pople, *J. Chem. Phys.*, 1980, **72**, 650.
25. A. D. McLean and G. S. Chandler, *J. Chem. Phys.*, 1980, **72**, 5639.
26. A. D. Becke, *Physical Review A*, 1988, **38**, 3098.
27. C. Lee, W. Yang and R. G. Parr, *Physical Review B*, 1988, **37**, 785.
28. B. Miehlich, A. Savin, H. Stoll and H. Preuss, *Chem. Phys. Lett.*, 1989, **157**, 200.
39. K. Raghavachari and G. W. Trucks, *J. Chem. Phys.*, 1989, **91**, 1062.
30. R. C. Binning and L. A. Curtiss, *J. Comput. Chem.*, 1990, **11**, 1206.
31. M. P. McGrath and L. Radom, *J. Chem. Phys.*, 1991, **94**, 511.
32. A. D. Becke, *J. Chem. Phys.*, 1993, **98**, 5648.
33. J.-P. Blaudeau, M. P. McGrath, L. A. Curtiss and L. Radom, *J. Chem. Phys.*, 1997, **107**, 5016.
34. E. D. Glendening, A. E. Reed, J. E. Carpenter and F. Weinhold, *NBO Version 3.1*.
